# DNA ligase I fidelity mediates the mutagenic ligation of pol β oxidized and mismatch nucleotide insertion products in base excision repair

**DOI:** 10.1016/j.jbc.2021.100427

**Published:** 2021-02-16

**Authors:** Pradnya Kamble, Kalen Hall, Mahesh Chandak, Qun Tang, Melike Çağlayan

**Affiliations:** Department of Biochemistry and Molecular Biology, University of Florida, Gainesville, Florida, USA

**Keywords:** base excision repair, DNA ligase I, DNA polymerase β, aprataxin, flap Endonuclease 1, 8-oxodGTP, 2'-deoxyribonucleoside 5'-triphosphate, AOA1, oculomotor apraxia type 1, APTX, aprataxin, BER, base excision repair, BLI, biolayer interferometry, BSA, bovine serum albumin, dGTP, guanine in the nucleotide pool, EE/AA, E346A/E592A, FEN1, flap endonuclease, LIG, DNA ligase, pol, polymerase

## Abstract

DNA ligase I (LIG1) completes the base excision repair (BER) pathway at the last nick-sealing step after DNA polymerase (pol) β gap-filling DNA synthesis. However, the mechanism by which LIG1 fidelity mediates the faithful substrate–product channeling and ligation of repair intermediates at the final steps of the BER pathway remains unclear. We previously reported that pol β 8-oxo-2'-deoxyribonucleoside 5'-triphosphate insertion confounds LIG1, leading to the formation of ligation failure products with a 5'-adenylate block. Here, using reconstituted BER assays *in vitro*, we report the mutagenic ligation of pol β 8-oxo-2'-deoxyribonucleoside 5'-triphosphate insertion products and an inefficient ligation of pol β Watson–Crick–like dG:T mismatch insertion by the LIG1 mutant with a perturbed fidelity (E346A/E592A). Moreover, our results reveal that the substrate discrimination of LIG1 for the nicked repair intermediates with preinserted 3'-8-oxodG or mismatches is governed by mutations at both E346 and E592 residues. Finally, we found that aprataxin and flap endonuclease 1, as compensatory DNA-end processing enzymes, can remove the 5'-adenylate block from the abortive ligation products harboring 3'-8-oxodG or the 12 possible noncanonical base pairs. These findings contribute to the understanding of the role of LIG1 as an important determinant in faithful BER and how a multiprotein complex (LIG1, pol β, aprataxin, and flap endonuclease 1) can coordinate to prevent the formation of mutagenic repair intermediates with damaged or mismatched ends at the downstream steps of the BER pathway.

Human DNA ligases (LIGs) (LIG1, LIG3, and LIG4) catalyze the formation of a phosphodiester bond between the 5'-phosphate (P) and 3'-hydroxyl (OH) termini of a DNA intermediate during DNA replication, repair, and genetic recombination (1, 2, 3, 4). The DNA ligation reaction by a LIG includes three chemical sequential steps and requires ATP and a divalent metal ion (Mg^2+^) (5). In the first step, ATP is hydrolyzed to produce an adenylate (AMP) that is then covalently linked to the ligase active site lysine, forming the adenylated ligase (6). Next, after binding of the adenylated ligase to a nicked DNA substrate, the AMP group is transferred to the 5'-P end of the nick, forming an adenylated DNA intermediate (7). In the final step, LIG catalyzes a nucleophilic attack of the 3'-OH in the DNA nick on the adenylated 5'-P to form a phosphodiester bond (8). Despite the fact that the ligation mechanism is a universally conserved process, we still lack the understanding of how LIG recognizes and processes damaged or mismatched DNA ends.

Successful DNA ligation relies on the formation of a Watson–Crick base pair of the nicked DNA that is formed during prior gap-filling DNA synthesis by a DNA polymerase (9, 10). Human DNA polymerases and LIGs have been considered as key determinants of genome integrity (11). In our previous studies, we demonstrated the importance of the coordination between DNA polymerase (pol) β and LIG1 during the repair of single DNA base lesions through base excision repair (BER) (9, 10, 12, 13, 14, 15, 16). BER is a critical process for preventing the mutagenic and lethal consequences of DNA damage that arises from endogenous and environmental agents and underlies disease and aging (17, 18). The repair pathway involves a series of sequential enzymatic steps that are tightly coordinated through protein–protein interactions in a process referred to as passing-the-baton or substrate-product channeling (19, 20, 21). This mechanism includes hand off of repair intermediates from gap-filling DNA synthesis by pol β to the DNA ligation reaction by LIG1 at the downstream steps of the BER pathway (9). However, how deviations in the functional coordination between pol β and LIG1 affect the BER process remains largely unknown. In particular, abnormalities created due to incorporation of damaged or mismatch nucleotides by pol β could lead to the formation of toxic and mutagenic repair intermediates that can drive genome instability or cell death. For example, endogenous and exogenous oxidative stress can oxidize guanine in the nucleotide pool (dGTP), resulting in the formation of the most abundant form of oxidative DNA damage, that is, the nucleotide triphosphate, 2'-deoxyribonucleoside 5'-triphosphate (8-oxodGTP) (22, 23). The deleterious effect of 8-oxodGTP is mediated through its incorporation into the genome by repair or replication DNA polymerases (24). For example, pol β performs mutagenic repair by inserting 8-oxodGTP opposite adenine within an active site that exhibits a frayed structure because of the lack of base pairing with a template base after the oxidized nucleotide insertion (25). In our prior studies, we demonstrated that pol β 8-oxodGTP insertion confounds the DNA ligation step of the BER pathway and leads to the formation of a ligation failure product with a 5'-adenylate (AMP) block (12, 13, 14). We also reported the effect of pol β mismatch nucleotide insertion on the substrate-product channeling to LIG1 during the final steps of BER and the fidelity of this mechanism in the presence of the epigenetically important 5-methylcytosine base modifications (15, 16).

The X-ray crystal structures of the three human LIGs in complex with DNA have revealed a conserved three-domain architecture that encircles the nicked DNA and induce partial unwinding and alignment of the 3'- and 5'-DNA ends (26, 27, 28, 29, 30, 31, 32). Recently, the crystal structures of LIG1 revealed that the enzyme’s high fidelity is mediated by Mg^2+^-dependent DNA binding (referred as Mg^HiFi^ metal site), a strategy that the enzyme uses during the adenyl transfer and nick-sealing steps of the ligation reaction, and is scaffolded by the two conserved amino acid residues (E346 and E592) (32). Moreover, mutations at these glutamate residues to alanine (E346A/E592A or EE/AA) lead to an LIG1 enzyme with lower fidelity and create an open cavity that accommodates a damaged DNA terminus at the protein active site (32). However, how such a distinct environment, which the staggered LIG1 conformation creates due to mutations at the Mg^HiFi^ metal site, affects ligase substrate discrimination and coordination with pol β during substrate–product channeling of repair intermediates with damaged or mismatched DNA ends at the downstream steps of BER remains entirely undefined.

Aprataxin (APTX), a member of the histidine triad superfamily, hydrolyzes an adenylate (AMP) moiety from the 5'-end of DNA ligation failure products and allows further attempts at completing repair (33). In our previous studies, we reported a role of flap endonuclease 1 (FEN1) for the processing of blocked repair intermediates with 5'-AMP (34, 35, 36). This compensatory mechanism is important to complement a deficiency in APTX activity, which is associated with mutations in the *aptx* gene and linked to the autosomal recessive neurodegenerative disorder ataxia with oculomotor apraxia type 1 (AOA1) (37). Recently, the process by which APTX removes 5'-AMP during the ligation of oxidative DNA damage-containing DNA ends has been suggested as a surveillance mechanism that protects LIG1 from ligation failure (32). Yet, the specificity of APTX and FEN1 for the repair intermediates including 5'-AMP and 3'-damaged or mismatched ends that mimic the ligation failure products after pol β–mediated mutagenic 8-oxodGTP or mismatch insertion is still unknown.

In this study, we examined the importance of LIG1 fidelity for faithful substrate–product channeling and ligation of repair intermediates at the final steps of the BER pathway. For this purpose, we evaluated the LIG1 mutants with lower fidelity (E346A, E592A, and EE/AA) for the ligation of pol β nucleotide insertion products and the nicked DNA substrates with 3'-preinserted damaged or mismatched bases *in vitro*. Our findings revealed the mutagenic ligation of pol β 8-oxodGTP insertion products and an inefficient DNA ligation after pol β Watson–Crick–like dGTP:T mismatch insertion by the LIG1 EE/AA mutant in a reconstituted BER reaction. Moreover, the ligation efficiency of the nicked repair intermediates with preinserted 3'-8-oxodG or mismatches was found to be dependent on the type of the template base and requires the presence of a double mutation at the E346 and E592 residues that typically ensure high fidelity. Finally, our findings demonstrated the compensatory roles of the DNA-end trimming enzymes, APTX and FEN1, for the processing of the ligation failure products with a 5'-AMP in the presence of 8-oxodG or all 12 possible mismatched bases at the 3'-end of the mutagenic repair intermediate. The findings herein contribute to our understanding of the efficiency and fidelity of substrate–product channeling during the final steps of BER in situations involving aberrant LIG1 fidelity and provide novel insight into the importance of the coordinated repair by a multiprotein complex for faithful BER.

## Results

### Low-fidelity LIG1 stimulates the mutagenic ligation of pol β 8-oxodGTP insertion products

**Figure 1:**
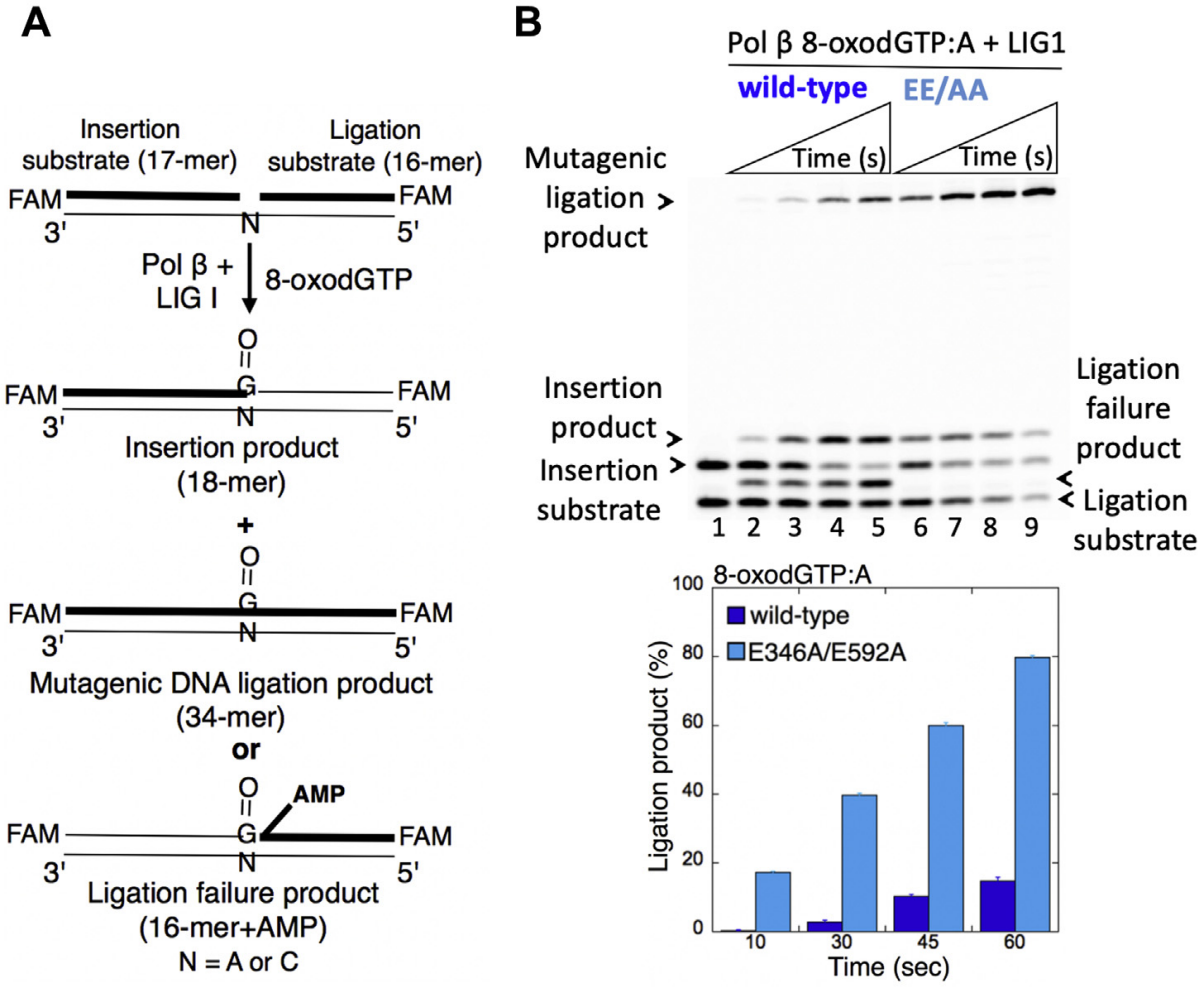
**Mutagenic ligation of pol β 8-oxodGTP insertion opposite A by low-fidelity LIG1.***A*, illustrations of the one-nucleotide-gap DNA substrate and the products of insertion, ligation, and ligation failure obtained in the coupled reactions. *B*, lane 1 is the negative enzyme control of the one-nucleotide-gap DNA substrate with template base A. Lanes 2 to 5 and 6 to 9 show the insertion coupled to ligation products by LIG1 WT and E346A/E592A (EE/AA) mutant, respectively, obtained at the time points 10, 30, 45, and 60 s. *C*, the graph shows the time-dependent changes in the ligation products, and the data are presented as the averages from three independent experiments ± SDs. 8-oxodGTP, 2'-deoxyribonucleoside 5'-triphosphate; FAM, 6-carboxyfluorescein; LIG1, DNA ligase I.

We previously demonstrated that the nicked repair product of pol β 8-oxodGTP insertion cannot be used as a DNA substrate by LIG1 during substrate–product channeling at the downstream steps of BER (12, 13, 14, 15, 16). In the present study, we first evaluated the effect of low-fidelity LIG1 for the ligation of pol β 8-oxodGTP insertion products *in vitro*. For this purpose, we used the coupled assay that measures the activities of pol β and LIG1 simultaneously in a reaction mixture that includes LIG1 WT or EE/AA mutant, pol β, 8-oxodGTP, and one-nucleotide-gap DNA substrate with template base A or C (Fig. 1*A*).

For the one-nucleotide-gap DNA substrate with template A, consistent with our previous studies (13, 15), we observed that WT LIG1 fails after pol β 8-oxodGTP insertion opposite A (Fig. 1*B*, lanes 2–5). This feature of LIG1 results in ligation failure and accumulation of abortive ligation products with 5'-adenylate (5'-AMP). The observed ligation failure was accompanied by the formation of mutagenic ligation (*i.e.*, sealing of the 3'-damage-containing nick intermediate, in this case, 8-oxodG) over the time of reaction incubation (Fig. 1*C*). Conversely, there was no ligation failure after pol β oxidized nucleotide insertions in the presence of the LIG1 mutant EE/AA that impairs the ligase fidelity. In this case, we only observed the products of mutagenic ligation after the pol β 8-oxodGTP insertion opposite A (Fig. 1*B*, lanes 6–9). The amount of this mutagenic ligation product was ∼10-fold higher than that of the product obtained with WT LIG1 (Fig. 1*C*).

**Figure 2:**
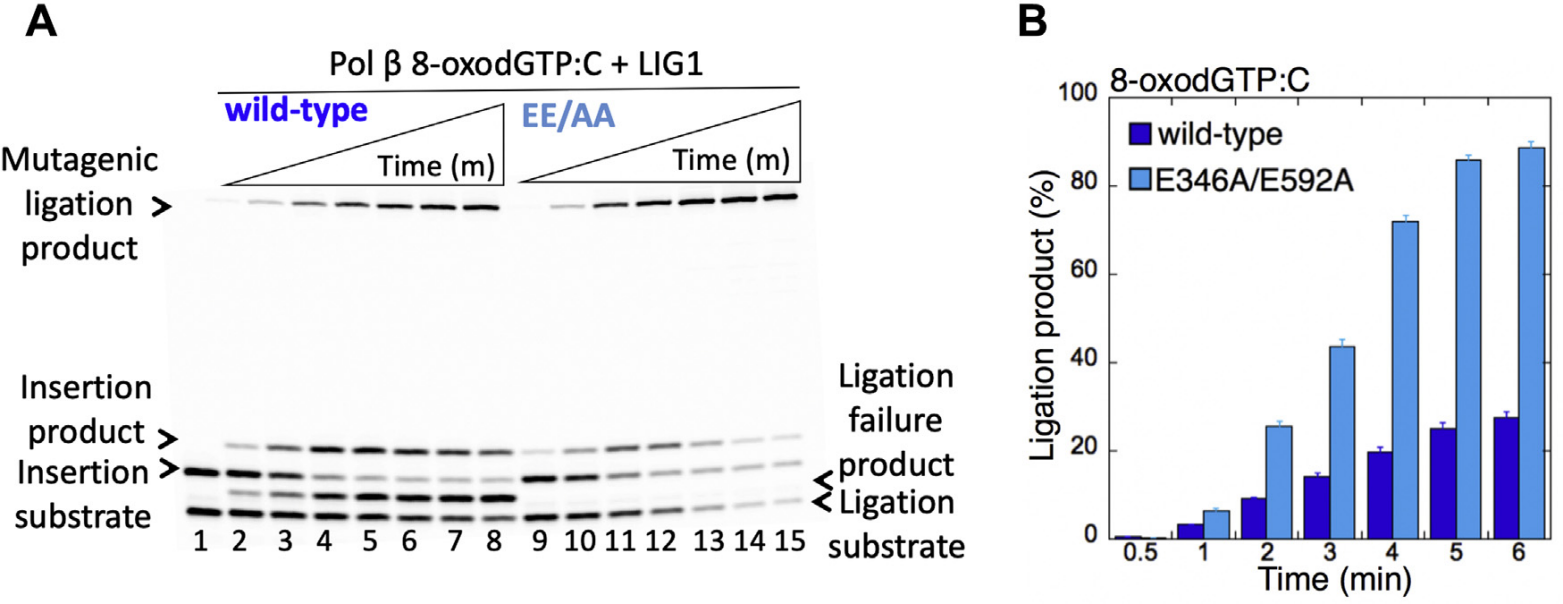
**Mutagenic ligation of pol β 8-oxodGTP insertion opposite C by low-fidelity LIG1.***A*, lane 1 is the negative enzyme control of the one-nucleotide-gap DNA substrate with template base C. Lanes 2 to 8 and 9 to 15 show the insertion coupled to ligation products by LIG1 WT and E346A/E592A (EE/AA) mutant, respectively, obtained at the time points 0.5, 1, 2, 3, 4, 5, and 6 min. *B*, the graph shows the time-dependent changes in the ligation products, and the data are presented as the averages from three independent experiments ± SDs. 8-oxodGTP, 2'-deoxyribonucleoside 5'-triphosphate; LIG1, DNA ligase I.

For the one-nucleotide-gap DNA substrate with template C, we also obtained the products of mutagenic ligation and ligation failure by WT LIG1 after pol β 8-oxodGTP insertion opposite C (Fig. 2*A*, lanes 2–8). However, due to the pol β weak insertion efficiency of 8-oxodGTP:C as reported previously (13, 15), the mutagenic ligation and 5'-adenylate products accumulated at later time points of the reaction (2–6 min) compared with the products obtained at earlier time points (10–60 s) in the case of pol β 8-oxodGTP:A insertion (Fig. 1*B* *versus* Fig. 2*B*). Similarly, we observed only the products of mutagenic ligation in the coupled reactions that include the LIG1 low-fidelity mutant EE/AA (Fig. 2*A*, lanes 9–15). The amount of this mutagenic ligation product was also higher than that of the WT protein (Fig. 2*B*). Yet, the EE/AA mutant was ∼80-fold more efficient for ligation of the pol β product after 8-oxodGMP insertion opposite A relative to C for the initial time points (30 s and 60 s) that are common to both pol β insertion reactions (Fig. S1).

**Figure 3:**
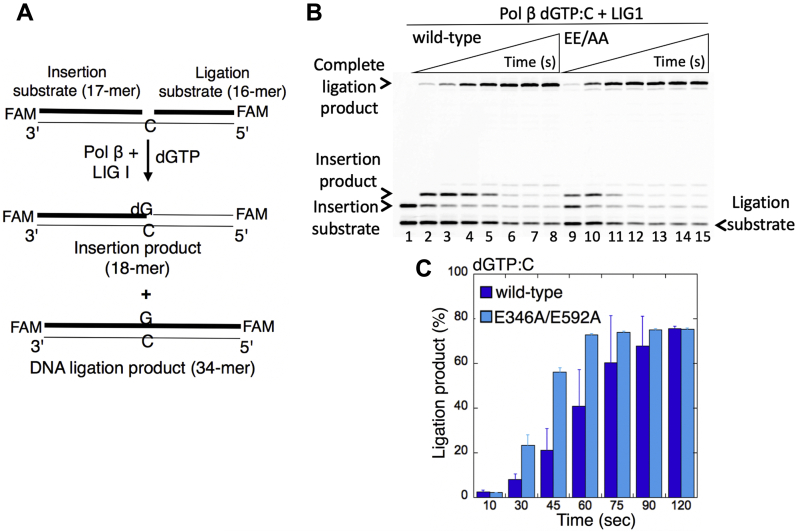
**Ligation of pol β dGTP insertion opposite C by low-fidelity LIG1.***A*, illustrations of the one-nucleotide-gap DNA substrate and the products of insertion and ligation obtained in the control-coupled reactions. *B*, lane 1 is the negative enzyme control of the one-nucleotide-gap DNA substrate with template base C. Lanes 2 to 8 and 9 to 15 show the insertion coupled to ligation products by LIG1 WT and E346A/E592A (EE/AA) mutant, respectively, obtained at the time points 10, 30, 45, 60, 75, 90, and 120 s. *C*, the graph shows the time-dependent changes in the products of insertion and ligation. The data are presented as the averages from three independent experiments ± SDs. dGTP, guanine in the nucleotide pool; LIG1, DNA ligase I; pol, polymerase.

In the control coupled reactions that include pol β, dGTP, and one-nucleotide-gap DNA substrate with template C, we evaluated the ligation of pol β dGTP:C insertion products in the presence of either WT protein or the LIG1 mutant EE/AA (Fig. 3*A*). The results demonstrated a complete ligation product over the time of reaction for both WT and the low-fidelity LIG1 mutant (Fig. 3*B*, lanes 2–8 and 9–15, respectively). In this case, we did not observe a significant difference in the amount of ligation products between WT and the EE/AA mutant (Fig. 3*C*). We then compared the efficiency of pol β nucleotide insertion and its conversion to the ligation products by WT or EE/AA mutant in the insertion (pol β alone) and coupled (pol β and LIG1) reactions separately (Fig. S2). The experiments demonstrated a more efficient conversion of pol β dGTP:C insertions to ligated repair products in the presence of the EE/AA mutant as shown by a faster decrease in the amount of the insertion products at the same time points of both reactions (Fig. S2, *A* and *B*). Overall results indicate that (i) the functional coordination between pol β and LIG1 is sensitive to the ligase fidelity and (ii) the cavity that is formed due to the EE/AA mutation at the Mg^HiFi^ of the LIG1 active site facilitates sealing of the nicked pol β repair product including an inserted 8-oxodGMP, while showing slight differences in the efficiency of mutagenic ligation depending on the type of template base to which pol β inserts the damaged nucleotide (Scheme S1).

### Ligation failure of pol β 8-oxodGTP insertion products by LIG1 deficiency disease variants

The mutations (P529L, E566K, R641L, and R771W) in the *LIG1* gene have been identified in the patients with LIG1-deficiency syndrome that exhibit immunodeficiency and cancer predisposition (38, 39). These amino acid residues residing in the different domains of the protein are located apart from the Mg^HiFi^ (E346 and E592) at the LIG1 active site (Fig. S3). Recently, we reported that the LIG1 variants associated with LIG1-deficiency disease exhibit altered ligation fidelity for pol β–promoted mutagenesis products (14). In the present study, in addition to the LIG1 low-fidelity mutants, we evaluated the effect of the LIG1 variants for the ligation of pol β 8-oxodGTP insertion products *in vitro* using the same coupled assay as described above.

**Figure 4:**
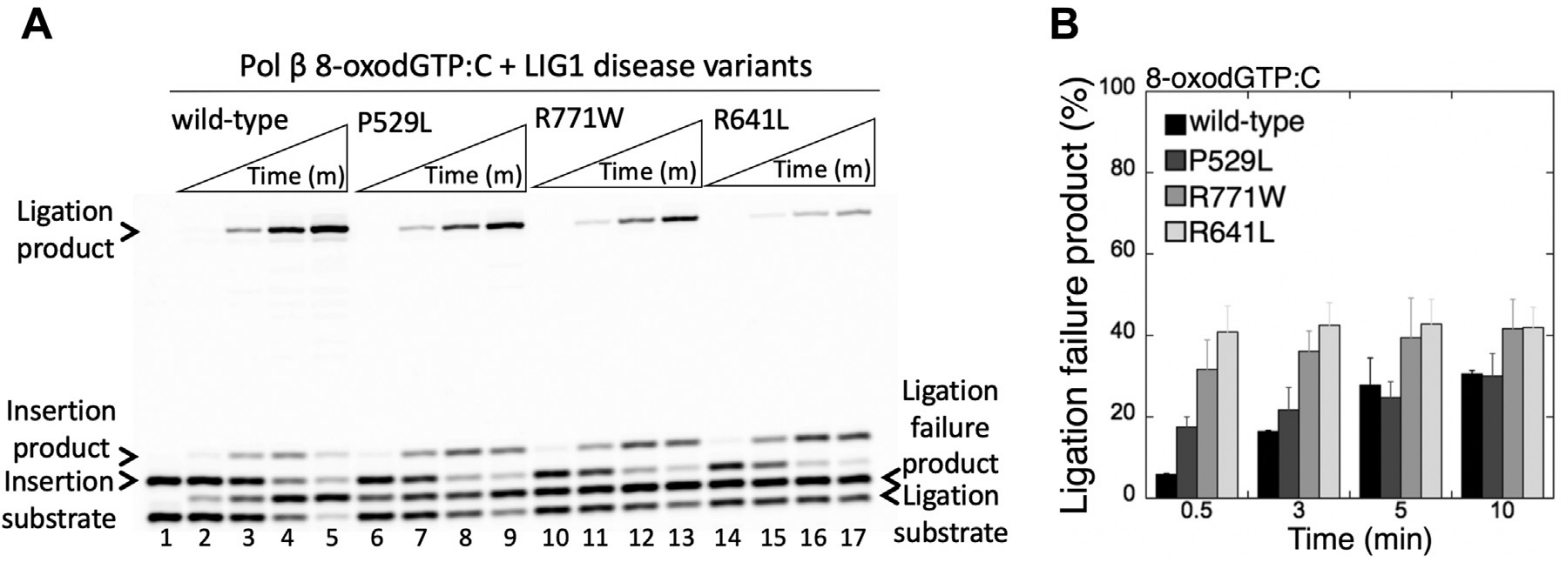
**Mutagenic ligation of pol β 8-oxodGTP insertion opposite C by LIG1 deficiency disease variants.***A*, lane 1 is the negative enzyme control of the one-nucleotide-gap DNA substrate with template base C. Lanes 2 to 5, 6 to 9, 10 to 13, and 14 to 17 show the insertion coupled to ligation products by LIG1 WT, P529L, R771W, and R641L variants, respectively, obtained at the time points 0.5, 3, 5, and 10 min. *B*, the graph shows the time-dependent changes in the ligation failure products, and the data are presented as the averages from three independent experiments ± SDs. 8-oxodGTP, 2'-deoxyribonucleoside 5'-triphosphate; LIG1, DNA ligase I.

In contrast to the mutagenic ligation observed with the LIG1 EE/AA mutant (Figs. 1 and 2), we obtained the ligation failure after the pol β 8-oxodGTP insertions by all LIG1 variants tested in this study (Fig. 4). For example, the LIG1 variants P529L, R771W, and R641L fail on the pol β repair product with an inserted 8-oxodGMP opposite C (Fig. 4*A*, lanes 6–9, 10–13, and 14–17, respectively), which yielded the ligation failure products in a BER reaction as also shown for WT LIG1 (Fig. 4*A*, lanes 2–5). The amounts of ligation failure products by the variants were higher than that of the WT protein and exhibit slight differences between the disease mutations (Fig. 4*B*). These findings suggest that the disease-associated LIG1 variants (*i.e.*, P529L, R641L, and R771W) do not interfere with the ligase fidelity and exhibit WT level of ligation efficiency in preventing formation of mutagenic repair intermediates after pol β oxidized nucleotide insertions (Scheme S1). In the recently published study (40), the role of destabilized Mg^2+^ cofactor binding in the ligation failure by the LIG1 deficiency disease mutants that could contribute to the development of the disease pathology has been suggested.

### Mutagenic ligation of the nicked repair intermediates with preinserted 3'-8-oxodG by low-fidelity LIG1

**Figure 5:**
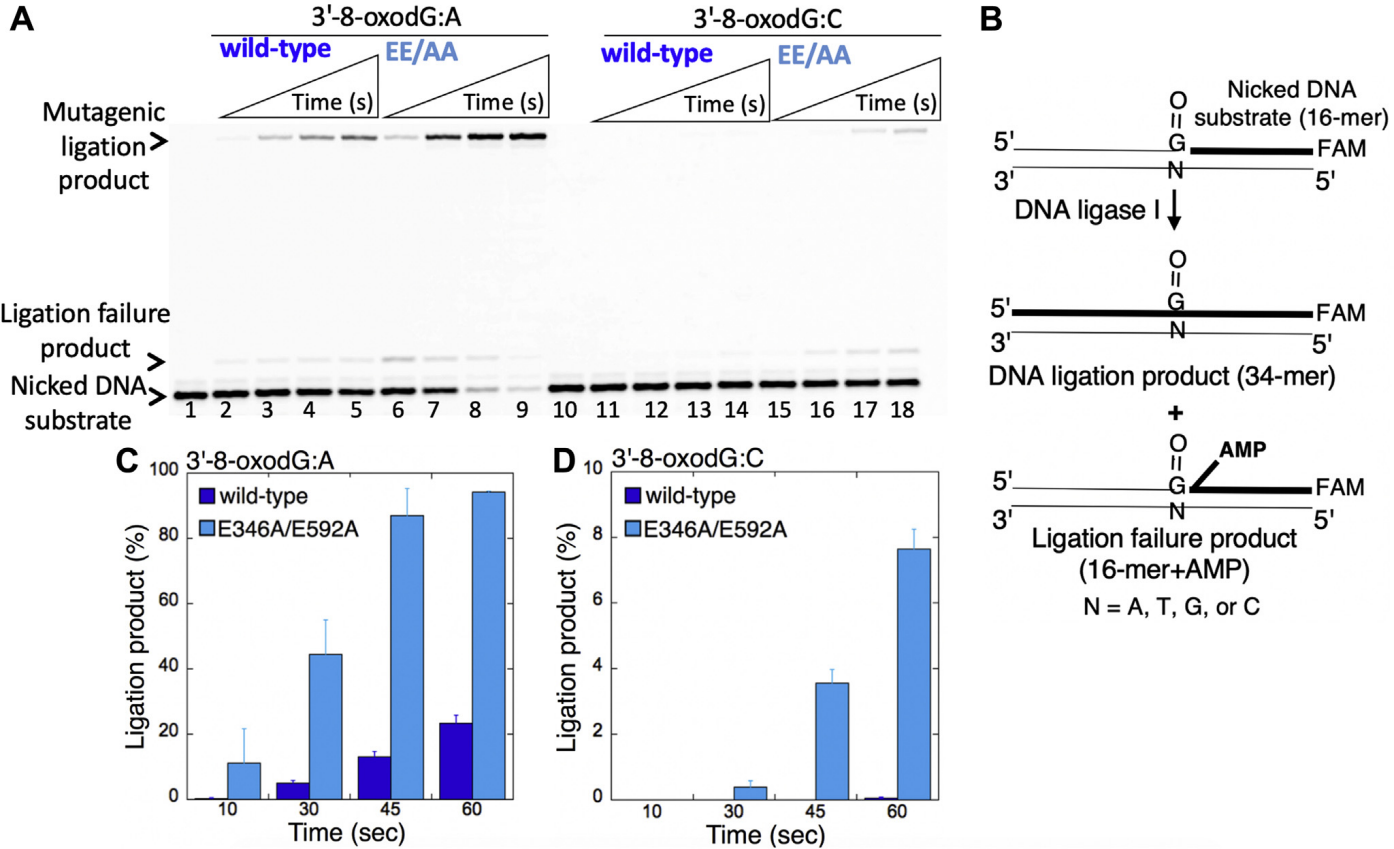
**Mutagenic ligation of the nicked DNA substrate with preinserted 3'-8-oxodG by low-fidelity LIG1.***A*, lanes 1 and 10 are the negative enzyme controls of the nicked DNA substrates with 3'-8-oxodG opposite template base A or C, respectively. Lanes 2 to 5 and 6 to 9 show the ligation products by LIG1 WT and E346A/E592A mutant, respectively, for 3'-8-oxodG:A substrate, obtained at the time points 10, 30, 45, and 60 s. Lanes 11 to 14 and 15 to 18 show the ligation products by LIG1 WT and E346A/E592A mutant, respectively, for 3'-8-oxodG:C substrate, obtained at the time points 10, 30, 45, and 60 s. *B*, illustrations of the nicked DNA substrate with 3'-8-oxodG and the products observed in the ligation reaction. *C*, the graphs show the time-dependent changes in the ligation products, and the data are presented as the averages from three independent experiments ± SDs. LIG1, DNA ligase I.

We then evaluated the importance of ligase fidelity on direct DNA end-joining of the repair intermediates that mimic DNA polymerase oxidized nucleotide (8-oxodGTP) insertion products *in vitro*. For this purpose, we used the ligation assay in a reaction mixture that includes either LIG1 WT or all low-fidelity mutants (E346A, E592A, or EE/AA) and the nicked DNA substrate that harbors a preinserted 3'-8oxodG (Fig. 5*B*).

For the nicked DNA substrate with 3'-8-oxodG opposite A, the results showed very efficient formation of mutagenic ligation products accumulated at earlier time points (10–60 s) by both WT and EE/AA mutants (Fig. 5*A*, lanes 2–5 and 6–9, respectively). This mutagenic ligation was ∼10-fold enhanced by the presence of the EE/AA mutation (Fig. 5*C*). However, consistent with our previous reports (12, 13, 14, 15, 16), LIG1 cannot efficiently ligate the nicked DNA substrate with 3'-8-oxodG opposite C at the earlier time points where we obtained relatively low amount of mutagenic ligation products by the WT LIG1 (Fig. 5*A*, lanes 11–14), which was also stimulated (∼8-fold) by the EE/AA mutant (Fig. 5*A*, lanes 15–18 and Fig. 5*D*).

We then tested the ligation efficiency of two single and one double LIG1 low-fidelity mutants (E346A, E592A, and EE/AA) for the repair intermediates that mimic pol β 8-oxodGTP insertion products opposite all possible template bases (A, T, G, or C). For these ligation reactions, we have chosen longer time points (0.5–10 min) because we were not able to observe ligation products for the nicked DNA substrates with preinserted 3'-8-oxodG opposite other three template bases when compared with the very efficient and fast mutagenic ligation of 3'-8-oxodG opposite A at earlier time points (10–60 s) of the reaction (Fig. 5 and Fig. S4).

**Figure 6:**
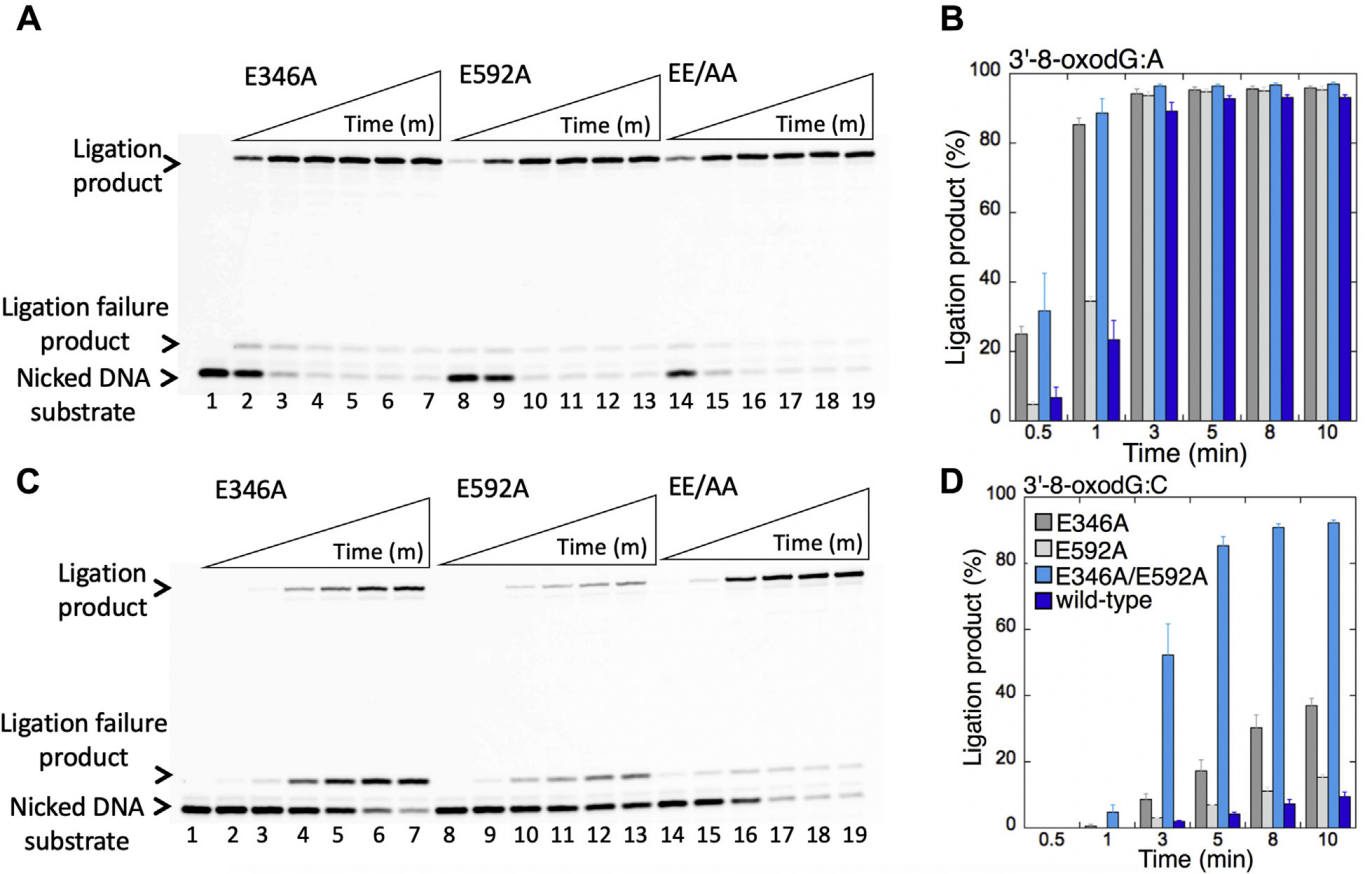
**Ligation efficiency of the repair intermediate with 3'-8-oxodG opposite A or C by low-fidelity LIG1 mutants.***A* and *C*, lane 1 is the negative enzyme control of the nicked DNA substrate with 3'-8-oxodG opposite template base A (*A*) or C (*C*). Lanes 2 to 7, 8 to 13, and 14 to 19 show the ligation products by LIG1 mutants E346A, E592A, and E346A/E592A (EE/AA), respectively, for 3'-8-oxodG:A (*A*) and 3'-8-oxodG:C (*C*) substrates, obtained at the time points 0.5, 1, 3, 5, 8, and 10 min. *B* and *D*, the graphs show the time-dependent changes in the ligation products, and the data are presented as the averages from three independent experiments ± SDs. LIG1, DNA ligase I.

For the nicked DNA substrate with preinserted 3'-8-oxodG opposite A, we did not observe a significant difference between LIG1 single mutants E346A (Fig. 6*A*, lanes 2–7), E592A (Fig. 6*A*, lanes 8–13), and the LIG1 double-mutant EE/AA (Fig. 6*A*, lanes 14–19) at longer time points of incubation, and the amount of mutagenic ligation products was similar (Fig. 6*B*). For the nicked DNA substrate with preinserted 3'-8-oxodG opposite C, we obtained more mutagenic ligation products in the presence of the LIG1 double-mutant EE/AA (Fig. 6*C*, lanes 14–19) than the ligation reactions with E346A (Fig. 6*C*, lanes 2–7) or E592A (Fig. 6*C*, lanes 8–13). There was an ∼80-fold difference in the amount of these ligation products between WT and the EE/AA mutant (Fig. 6*D*). In contrast to the nicked DNA with 3'-8-oxodG:A, we note that the mutagenic ligation of 3'-8-oxodG:C nicked DNA by low-fidelity mutants of LIG1 was accompanied by the formation of ligation failure products and we observed more ligation failure products in the presence of single low-fidelity mutants E346A and E592A than double EE/AA mutant (Fig. S5*A*).

**Figure 7:**
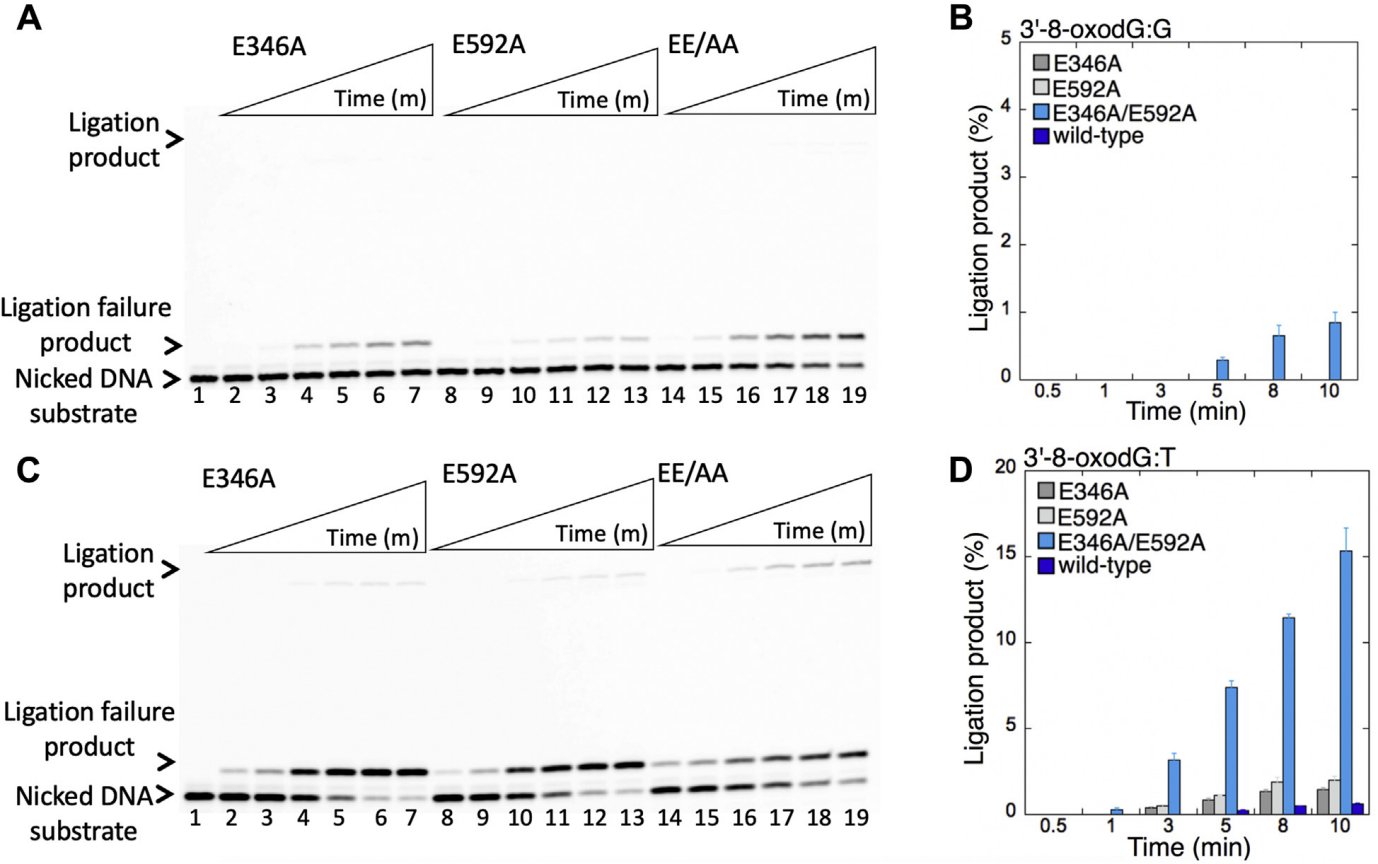
**Ligation efficiency of the repair intermediate with 3'-8-oxodG opposite G or T by low-fidelity LIG1 mutants.***A* and *C*, lane 1 is the negative enzyme control of the nicked DNA substrate with 3'-8-oxodG opposite template base G (*A*) or T (*C*). Lanes 2 to 7, 8 to 13, and 14 to 19 show the ligation products by LIG1 mutants E346A, E592A, and E346A/E592A (EE/AA), respectively, for 3'-8-oxodG:G (*A*) and 3'-8-oxodG:T (*C*) substrates, obtained at the time points 0.5, 1, 3, 5, 8, and 10 min. *B* and *D*, the graphs show the time-dependent changes in the ligation products, and the data are presented as the averages from three independent experiments ± SDs. LIG1, DNA ligase I.

Interestingly, for the nicked DNA substrates containing 3'-8-oxodG opposite G or T, we did not observe mutagenic ligation in the presence of any LIG1 mutant with perturbed fidelity (Fig. 7). There were only ligation failure products on nicked DNA with 3'-8-oxodG:G ends by LIG1 E346A (Fig. 7*A*, lanes 2–7), E592A (Fig. 7*A*, lanes 8–13), and EE/AA (Fig. 7*A*, lanes 14–19). Similarly, for the nicked DNA substrate with 3'-8-oxodG:T, we obtained a negligible amount of ligation products only by the EE/AA mutant (Fig. 7*D*). Conversely, the results showed the accumulation of very high amount of ligation failure products in the presence of E346A (Fig. 7*C*, lanes 2–7), E592A (Fig. 7*C*, lanes 8–13), and EE/AA (Fig. 7*C*, lanes 14–19) as revealed by the formation of the 5'-AMP products over the time of ligation reaction incubation. Similarly, there were more ligation failure products of 3'-8-oxodG:T nicked DNA in the presence of single low-fidelity mutants E346A and E592A than the double EE/AA mutant (Fig. S5*C*).

When compared with the ligation efficiency of WT LIG1 (Fig. S6), overall results indicate that the mutagenic ligation of the nicked repair intermediates that mimic the repair products of pol β 8-oxodGMP insertions is template base dependent and requires the mutations at both active site residues (E346 and E592) that enforce ligase fidelity (Fig. S7*A*). The ligation failure by the EE/AA mutant was only obtained when 8-oxodG is base-paired with G or T in a template position (Fig. S7*B*).

### Inefficient ligation of pol β Watson–Crick–like dG:T insertion products by low-fidelity LIG1

**Figure 8:**
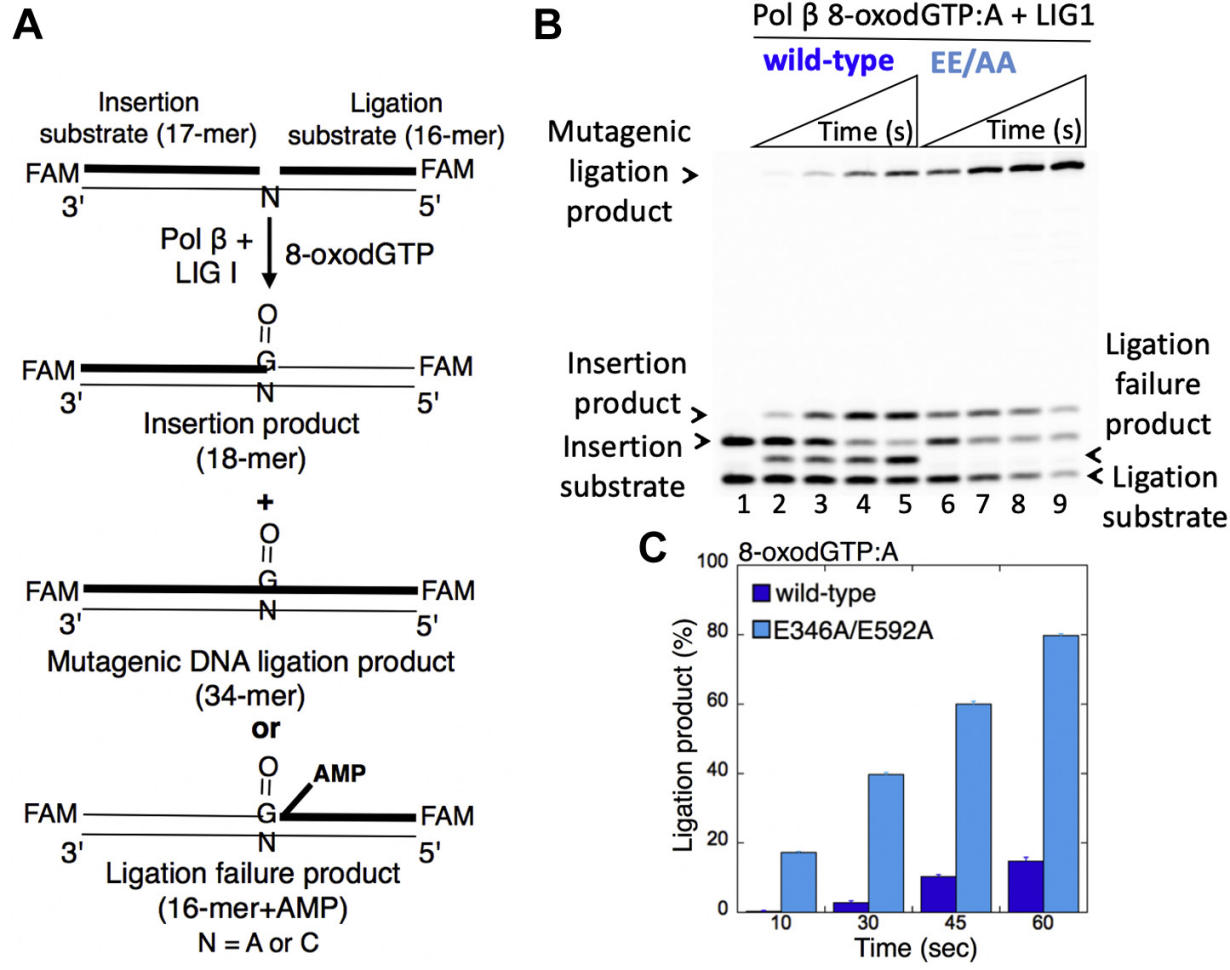
**Ligation of pol β dGTP insertion opposite T by low-fidelity LIG1**. *A*, illustrations of the one-nucleotide-gap DNA substrate and the products obtained in the coupled reaction. *B*, lane 1 is the positive control for the ligation of pol β correct dGTP:C insertion product by WT LIG1. Lane 2 is the negative enzyme control of the one-nucleotide-gap DNA substrate with template base T. Lanes 3 to 9 and 10 to 16 show the insertion coupled to ligation products by LIG1 WT and E346A/E592A (EE/AA) mutant, respectively, obtained at the time points 10, 30, 45, 60, 75, 90, and 120 s. The gel is a representative of three independent experiments. *C*, the graph shows the time-dependent changes in the ligation products. The data are presented as the averages from three independent experiments ±SDs. dGTP, guanine in the nucleotide pool; LIG1, DNA ligase I; pol, polymerase.

In our prior work, we reported that LIG1 efficiently ligates the pol β Watson–Crick–like dGTP:T insertion products (16). To further examine the effect of a staggered LIG1 conformation with perturbed fidelity on the substrate–product channeling of repair intermediates at the downstream steps of the BER pathway, we also evaluated the ligation of pol β dGTP:T insertion products in the coupled reaction including LIG1 WT or EE/AA mutant, pol β, dGTP, and the one-nucleotide-gap DNA substrate with template base T (Fig. 8*A*).

Surprisingly, in the reactions with the LIG1 mutant EE/AA, the main products were self-ligation products, where the two ends within the one-nucleotide-gap DNA were directly ligated (Fig. 8*B*, lanes 10–16). The conclusion about self-ligation was drawn based on the difference in the size of products obtained in comparison with those generated *via* the ligation of pol β dGTP:T mismatch insertion (Fig. 8*B*, lanes 3–9 *versus* lanes 10–16) or the ligation of pol β dGTP:C correct insertion (Fig. 8*B*, lane 1 *versus* lanes 10–16) products by WT LIG1.

The comparisons for the pol β dGTP:C *versus* dGTP:T insertions and their conversion to the ligated products in the insertion (pol β alone) and coupled (pol β and LIG1) reactions separately demonstrated a very low efficiency of pol β dGTP insertion opposite T (Fig. S2*C*, lanes 2–5), which yielded complete (albeit mutagenic) ligation products by WT LIG1 (Fig. S2*C*, lanes 7–10). However, these ligation products were accompanied by strong self-ligations of the one-nucleotide-gap DNA by the EE/AA mutant at the same time points of both reactions (Fig. S2*C*, lanes 11–14).

**Figure 9:**
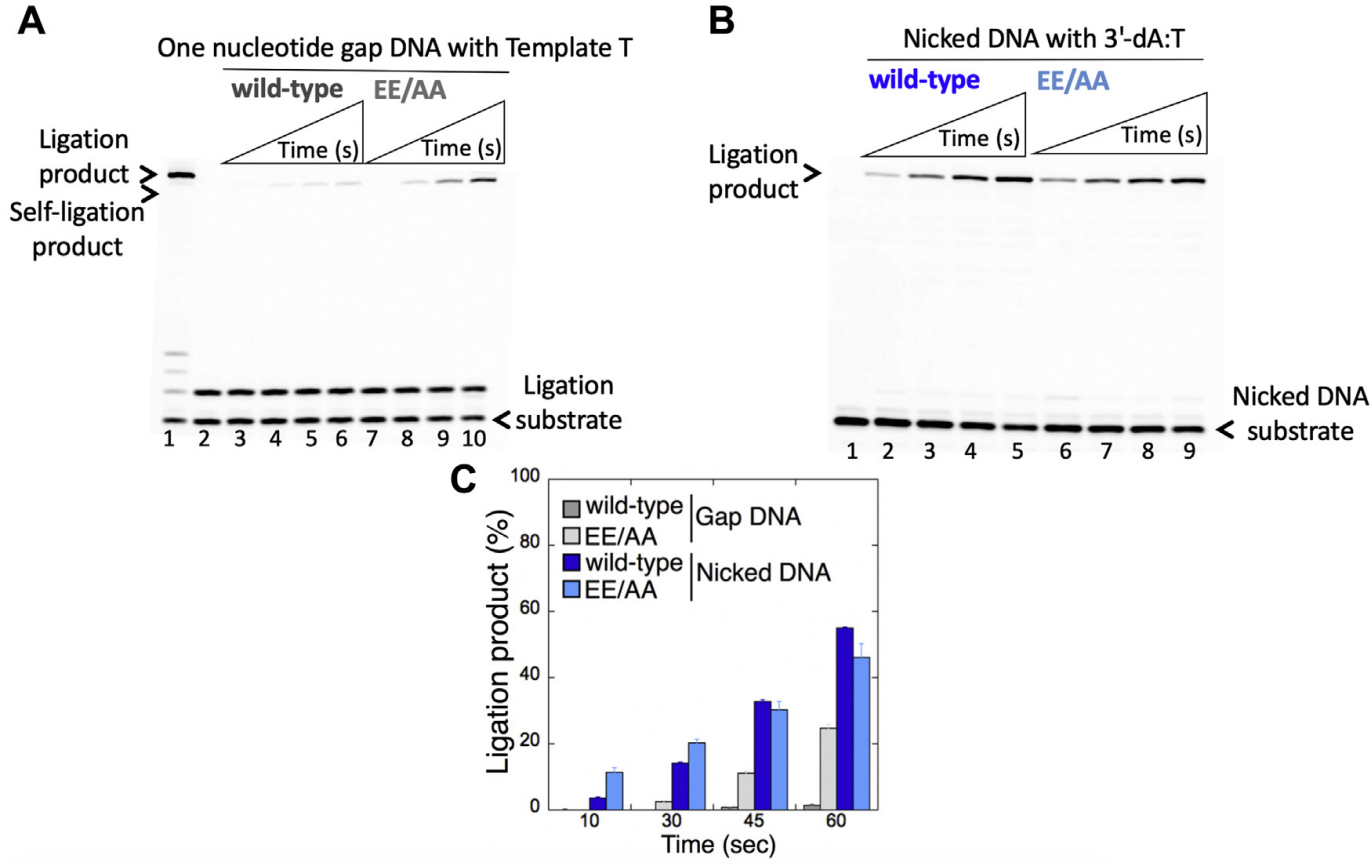
**Ligation efficiency of one-nucleotide-gap and nicked DNA substrates by low-fidelity LIG1.***A*, lane 1 is the positive control for the ligation of pol β correct dGTP:C insertion product by WT LIG1. Lane 2 is the negative enzyme control of the one-nucleotide-gap DNA substrate with template base T. Lanes 3 to 6 and 7 to 10 show the self-ligation products by LIG1 WT and E346A/E592A (EE/AA) mutant, respectively, obtained at the time points 10, 30, 45, and 60 s. *B*, lane 1 is the negative enzyme control of the nicked DNA substrate with 3'-dA:T. Lanes 2 to 5 and 6 to 9 show the ligation products by LIG1 WT and E346A/E592A mutant, respectively, obtained at the time points 10, 30, 45, and 60 s. *C*, the graph shows the time-dependent changes in the ligation of the nicked DNA *versus* self-ligation of gap DNA products, and the data are presented as the averages from three independent experiments ± SDs. dGTP, guanine in the nucleotide pool; LIG1, DNA ligase I.

We then investigated this self-ligation product further by comparing the ligation efficiency of one-nucleotide-gap DNA with template T *versus* the nicked DNA with preinserted 3'-dA:T (Fig. 9). The results demonstrated that WT LIG1 has stronger propensity to ligate the ends at the nick (Fig. 9*B*, lanes 2–5) over the gap (Fig. 9*A*, lanes 2–5), which shows ∼60-fold difference (Fig. 9*C*). However, this inclination was significantly diminished in the presence of the EE/AA mutant for the gap DNA (Fig. 9*A*, lanes 7–10) over the nick (Fig. 9*B*, lanes 7–10), which is now only ∼2-fold difference (Fig. 9*C*). Furthermore, using biolayer interferometry (BLI) analysis, we measured the real-time DNA-binding kinetics of LIG1 WT and EE/AA mutant for the gap DNA that was used in coupled reactions as described above. The results showed that the gap DNA binding affinity of the EE/AA mutant is relatively stronger (∼20 nM) than that of WT (∼83 nM) enzyme (Fig. S8). Overall results show that the LIG1 active site can accommodate the Watson–Crick–like G-T mismatch-containing DNA ends of pol β insertion product, while the EE/AA mutation at the Mg^HiFi^ site could result in sealing of gap DNA ends with template T to which pol β inserts a mismatched base (*i.e.*, dGTP) at a significantly lower efficiency (Fig. S2). We suggest that this pol β mismatch insertions or in case of any situation when pol β gap-filling activity and/or LIG1 fidelity is perturbed, the single base–deletion mutagenesis products could be formed because of the ligation of the gap repair intermediate at the downstream steps of the BER pathway (Scheme S1).

### The effect of aberrant LIG1 fidelity on DNA end-joining of 3'-preinserted noncanonical mismatches

We previously reported that the BER LIGs (LIG1 and LIG3) recognize subtle base differences at either the template or the 3'-end when sealing nicked DNA and that the mismatch discrimination of the ligases can vary depending on the DNA end structure of the repair intermediate (16). In the present study, we evaluated the 3'-end surveillance of low-fidelity LIG1 for ligation of 3'-preinserted mismatches that mimic pol β mismatch insertion products in the ligation reaction including the ligase alone.

**Figure 10:**
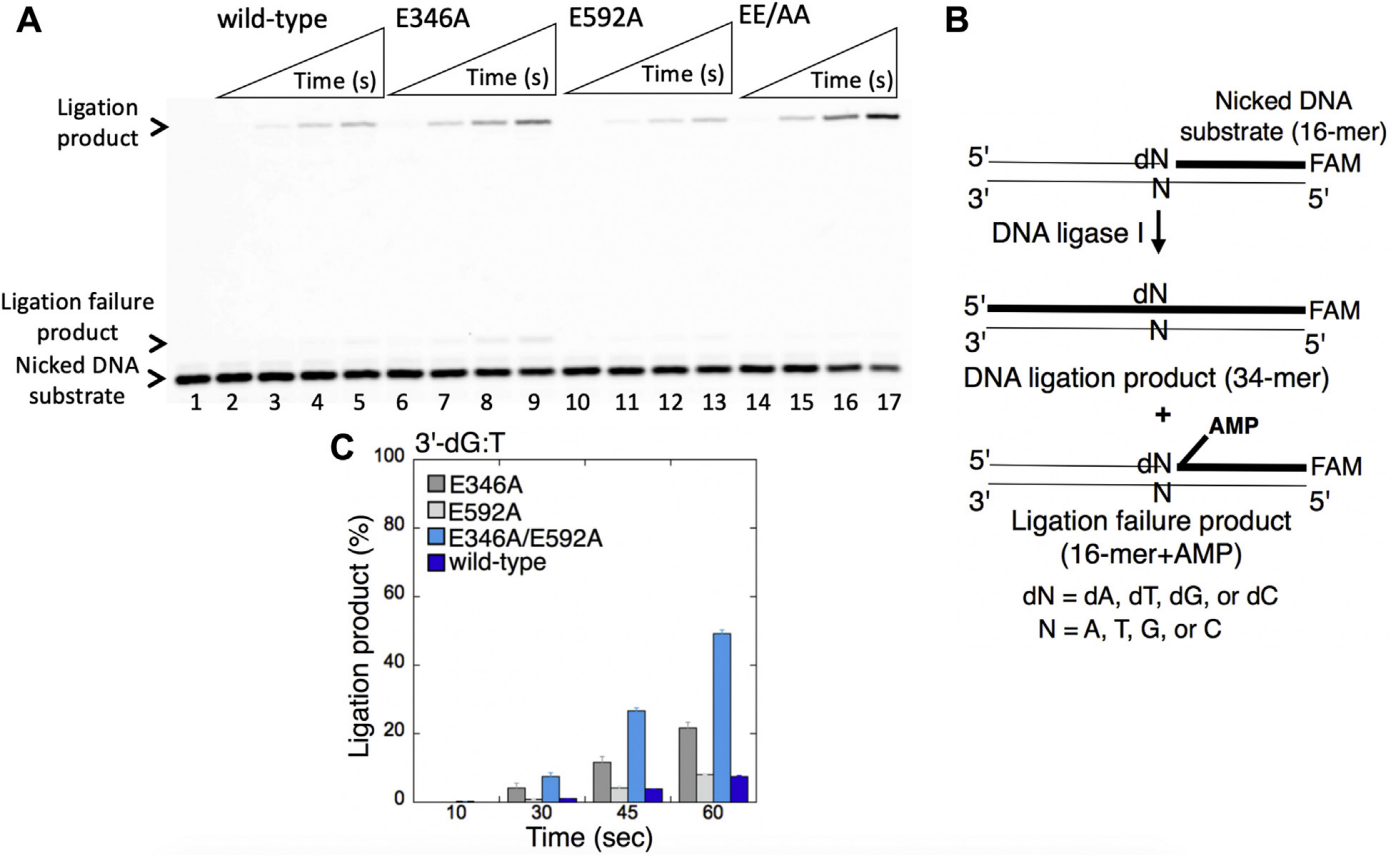
**Ligation efficiency of the repair intermediate with Watson–Crick–like 3'-dG:T mismatch by low-fidelity LIG1.***A*, lane 1 is the negative enzyme control of the nicked DNA substrate with 3'-dG:T mismatch, and lanes 2 to 5, 6 to 9, 10 to 13, and 14 to 17 show the ligation products by LIG1 WT, E346A, E592A, and E346A/E592A (EE/AA) mutants, respectively, obtained at the time points 10, 30, 45, and 60 s. *B*, illustrations of the nicked DNA substrate and the ligation and ligation failure products obtained in the reaction including preinserted 3'-mismatches. *C*, the graph shows the time-dependent changes in the ligation products, and the data are presented as the averages from three independent experiments ± SDs. LIG1, DNA ligase I.

We first tested the ligation of a nicked DNA substrate with preinserted Watson–Crick–like 3'-dG:T mismatch at the earlier time points (10–60 s) of reaction incubation (Fig. 10*B*). The results revealed mutagenic ligation of the nicked DNA substrate with 3'-dG:T mismatch by the WT LIG1 (Fig. 10*A*, lanes 2–5), which was also observed with low-fidelity LIG1 mutants E346A (Fig. 10*A*, lanes 6–9), E592A (Fig. 10*A*, lanes 10–13), and EE/AA (Fig. 10*A*, lanes 14–17). However, there was ∼50-fold increase in the amount of ligation products for the EE/AA mutant relative to WT LIG1 (Fig. 10*C*). For longer time points of ligation reaction (0.5–10 min), where we obtained strong ligation failure products only with the nicked DNA substrate including a damaged dG opposite T (3'-8oxodG:T, Fig. 7, *C* and *D*), the EE/AA mutant indeed showed a high amount of mutagenic ligation products (no ligation failure) for the nicked DNA with 3'-dG:T (Fig. S9, *A* and *B*). The difference in the efficiency of the LIG1 EE/AA low-fidelity mutant for the ligation of the repair intermediates with damaged (8-oxodG) *versus* undamaged (dG) base at 3'-end of nicked DNA with the same template base (T) (Fig. S9*C*) could be due to their distinct base-pairing features and the presence of oxygen modification on 8-oxodG with unfavorable synconformational/anticonformational equilibrium that may not be accommodated by the LIG1 active site as previously shown for DNA polymerases (41, 42, 43, 44, 45, 46).

**Figure 11:**
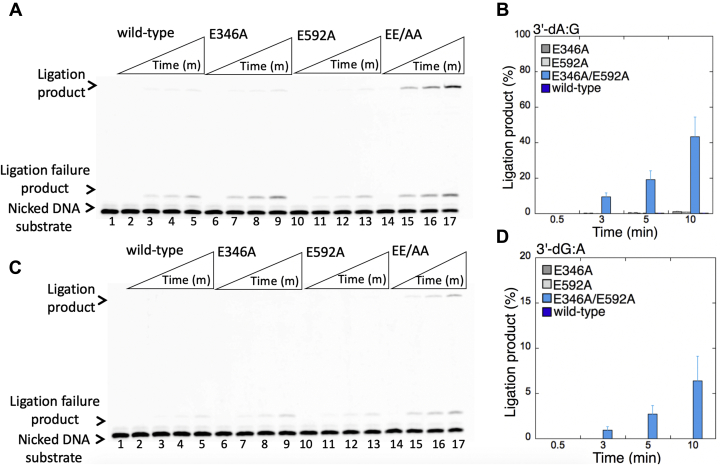
**Ligation efficiency of the repair intermediates with 3'-preinserted dA:G and dG:A mismatches by low-fidelity LIG1.***A* and *C*, lane 1 is the negative enzyme control of the nicked DNA substrate with 3'-dA:G (*A*) and 3'-dG:A (*C*) mismatches. Lanes 2 to 5, 6 to 9, 10 to 13, and 14 to 17 show the reaction products by LIG1 WT, E346A, E592A, and E346A/E592A (EE/AA) mutants, respectively, for 3'-dA:G (*A*) and 3'-dG:A (*C*) substrates, obtained at the time points 0.5, 3, 5, and 10 min. *B* and *D*, the graphs show the time-dependent changes in the ligation products, and the data are presented as the averages from three independent experiments ± SDs. LIG1, DNA ligase I.

**Figure 12:**
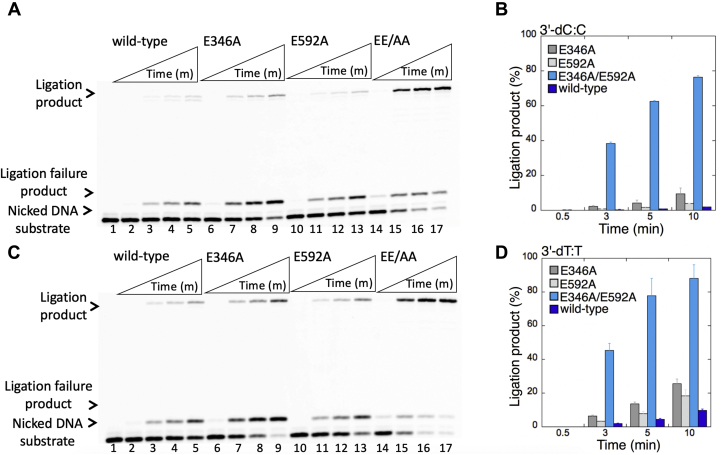
**Ligation efficiency of the repair intermediates with 3'-preinserted dC:C and dT:T mismatches by low-fidelity LIG1.***A* and *C*, lane 1 is the negative enzyme control of the nicked DNA substrate with 3'-dC:C (*A*) and 3'-dT:T (*C*) mismatches. Lanes 2 to 5, 6 to 9, 10 to 13, and 14 to 17 show the reaction products by LIG1 WT, E346A, E592A, and E346A/E592A (EE/AA) mutants, respectively, for 3'-dC:C (*A*) and 3'-dT:T (*C*) substrates, obtained at the time points 0.5, 3, 5, and 10 min. *B* and *D*, the graphs show the time-dependent changes in the ligation products, and the data are presented as the averages from three independent experiments ± SDs. LIG1, DNA ligase I.

We then tested the ligation efficiency of the nicked DNA substrates for which WT LIG1 exhibits compromised ligation, that is, 3'-preinserted dA:G, dG:A, dC:C, dT:T (Figs. 11 and 12). For the nicked substrates with DNA ends containing purine–purine base mismatches, 3'-dA:G and 3'-dG:A (Fig. 11), we did not observe ligation products by LIG1 single mutants, E346A (Fig. 11, *A* and *C*, lanes 6–9) and E592A (Fig. 11, *A* and *C*, lanes 10–13). This finding was similar to the inefficient ligation by WT LIG1 (Fig. 11, *A* and *C*, lanes 2–5). The presence of the EE/AA mutation (Fig. 11, *A* and *C*, lanes 14–17) slightly stimulated mutagenic ligation in comparison with the WT enzyme (Fig. 11, *B* and *D*). For the nicked substrates with DNA ends containing pyrimidine pairs, 3'-dC:C and 3'-dT:T (Fig. 12), in comparison with the inefficient ligation by WT LIG1 (Fig. 12, *A* and *C*, lanes 2–5), we observed significantly higher amount of mutagenic ligation products by LIG1 mutants E346A (Fig. 12, *A* and *C*, lanes 6–9), E592A (Fig. 12, *A* and *C*, lanes 10–13), and EE/AA (Fig. 12, *A* and *C*, lanes 14–17). For both pyrimidine DNA substrates, the presence of double mutation greatly enhanced mutagenic ligation, and there was ∼80-fold difference for both nicked DNA substrates in the amount of ligation products between WT and EE/AA (Fig. 12, *B* and *D*). However, we note that this mutagenic ligation has a certain requirement for the presence of both mutations at Mg^HiFi^ site as nick sealing of 3'-dT:T ends with single mutants (E346A or E592A) yielded very high amount of ligation failure products (Fig. S10). In the control ligation reactions containing the nicked DNA substrate with preinserted 3'-dG:C correctly base-paired ends, we confirmed the similar ligation efficiencies for the WT LIG1 and the low-fidelity mutants (Fig. S11). Overall, the results indicate that the mutagenic ligation of repair intermediates with preinserted 3'-mismatched bases by low-fidelity LIG1 is dependent on the architecture of DNA ends at the nick (Fig. S12). Similarly, structural studies have demonstrated that the pol β active site undergoes diverse mismatch-induced molecular adjustments, and the extent of these conformational distortions in the pol β active site is dependent on the architecture of the mismatched template primer (47, 48, 49, 50, 51).

### Roles of APTX and FEN1 in the processing of ligation failure intermediates with 5'-adenylate and 3'-8-oxodG or mismatch

We next examined the DNA end-processing proteins APTX and FEN1 for their functions to clean 5'-ends from the repair intermediates containing an adenylate (AMP) block that mimic the ligation failure products after pol β 8-oxodGTP or mismatch insertions. For this purpose, we evaluated the enzymatic activities of APTX and FEN1 in a reaction mixture that includes the nicked DNA substrates with preinserted 5'-AMP and 3'-preinserted 8-oxodG or one of the 12 possible noncanonical base pairs *in vitro*.

**Figure 13:**
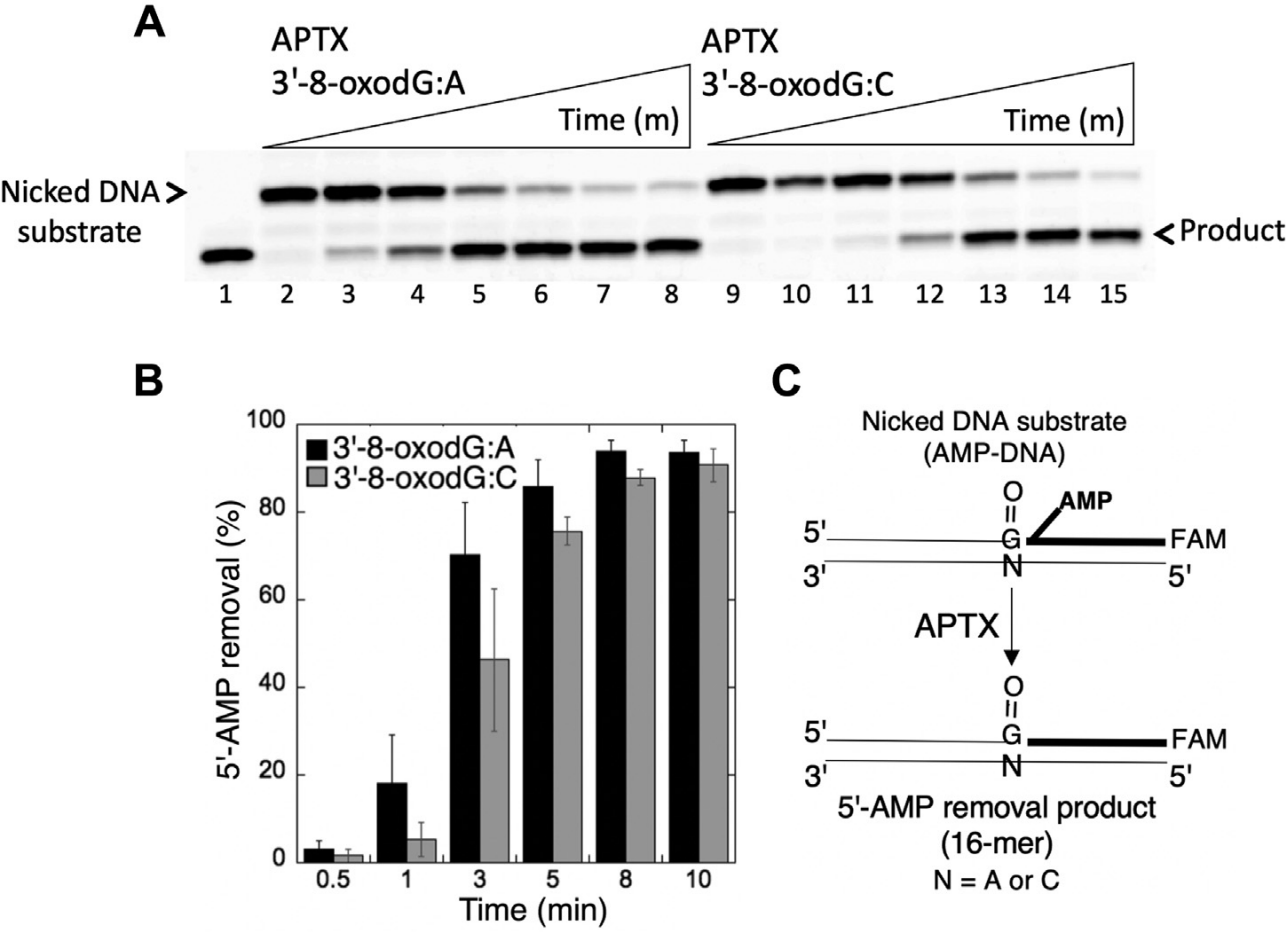
**Removal of 5'-AMP by APTX from the ligation failure products with 3'-8-oxodG.***A*, lane 1 is a size marker that corresponds to the oligonucleotide without an AMP moiety. Lanes 2 and 9 are the negative enzyme controls of the nicked DNA substrates with 5'-AMP and 3'-8-oxodG opposite template base A or C, respectively. Lanes 3 to 8 and 10 to 15 show the products of 5'-AMP removal from 3'-8-oxodG:A and 3'-8-oxodG:C substrates, respectively, obtained at the time points 0.5, 1, 3, 5, 8, and 10 min. *B*, the graph shows the time-dependent changes in the products of 5'-AMP removal, and the data are presented as the averages from three independent experiments ± SDs. *C*, illustrations of the nicked DNA substrate with 5'-AMP and 3'-8-oxodG and the products observed in the APTX reaction. APTX, aprataxin.

**Figure 14:**
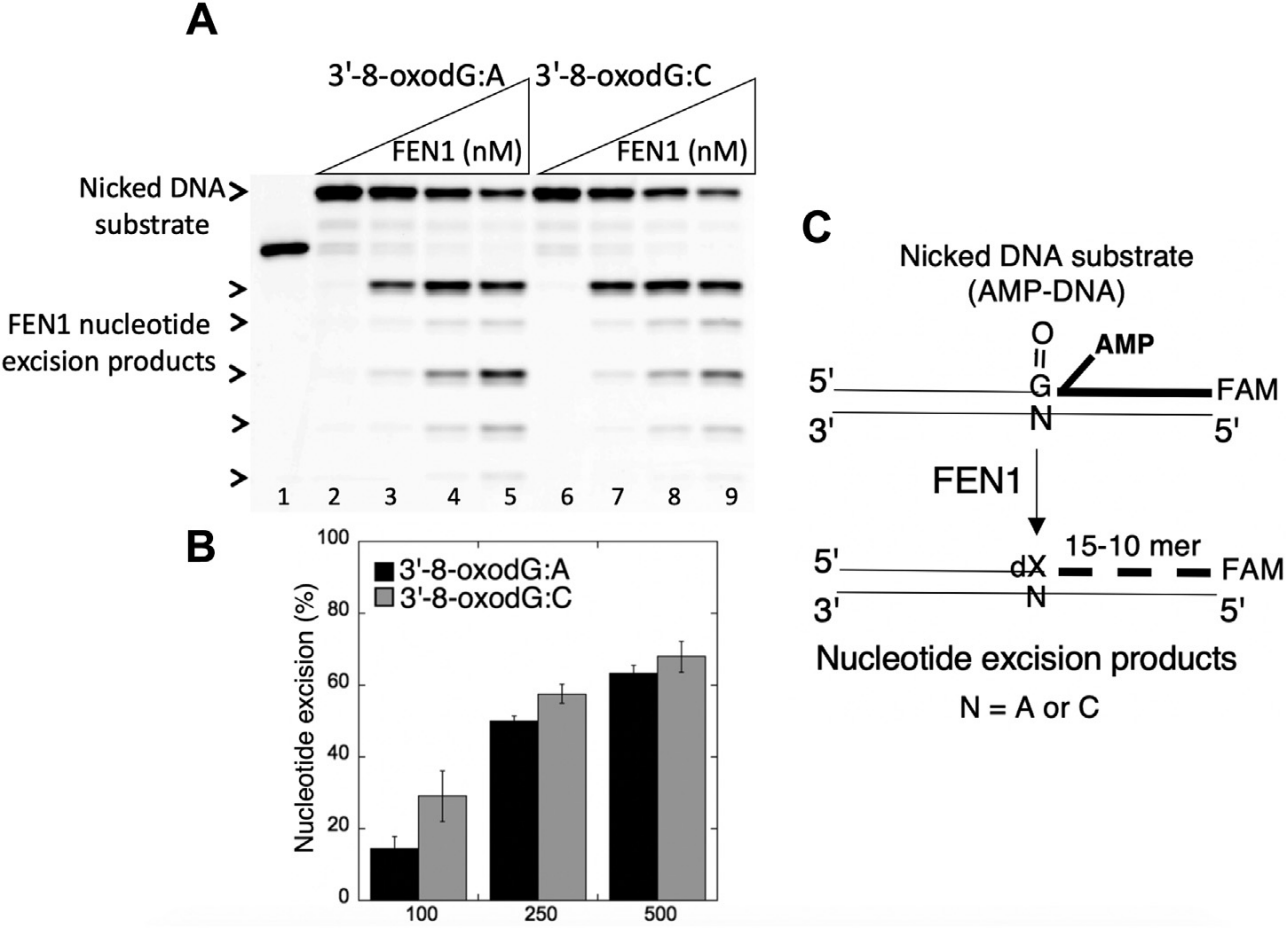
**The nucleotide excisions by FEN1 from the ligation failure products with 3'-8-oxodG.***A*, lane 1 is a size marker that corresponds to the oligonucleotide without an AMP moiety. Lanes 2 and 6 are the negative enzyme controls of the nicked DNA substrates with 5'-AMP and 3'-8-oxodG opposite template base A and C, respectively. Lanes 3 to 5 and 7 to 9 show the products of nucleotide excisions from 3'-8-oxodG:A and 3'-8-oxodG:C substrates, respectively, obtained as a function of FEN1 concentration. *B*, the graph shows the time-dependent changes in the products of nucleotide excisions, and the data are presented as the averages from three independent experiments ± SDs. *C*, illustrations of the nicked DNA substrate with 5'-AMP and 3'-8-oxodG, and the products observed in the FEN1 reaction. FEN1, flap endonuclease.

With the nicked DNA substrates with 5'-AMP and 3'-8-oxodG (Figs. 13*C* and 14*C*), we observed an efficient removal of the 5'-AMP block by APTX from DNA ends, such as 3'-8-oxodG opposite template base A (Fig. 13*A*, lanes 3–8) or template base C (Fig. 13*A*, lanes 10–15). The results showed no significant difference in the amount of 5'-AMP removal products from these two DNA substrates (Fig. 13*B*). For FEN1 activity, we obtained products representing both 5'-AMP removal and nucleotide excisions from the nicked DNA substrates containing 3'-8-oxodG opposite template base A (Fig. 14*A*, lanes 3–5) or template base C (Fig. 14*A*, lanes 7–9). The amount of nucleotide excision products showed no significant difference between the two substrates and increased as a function of FEN1 concentration (Fig. 14*B*).

**Figure 15:**
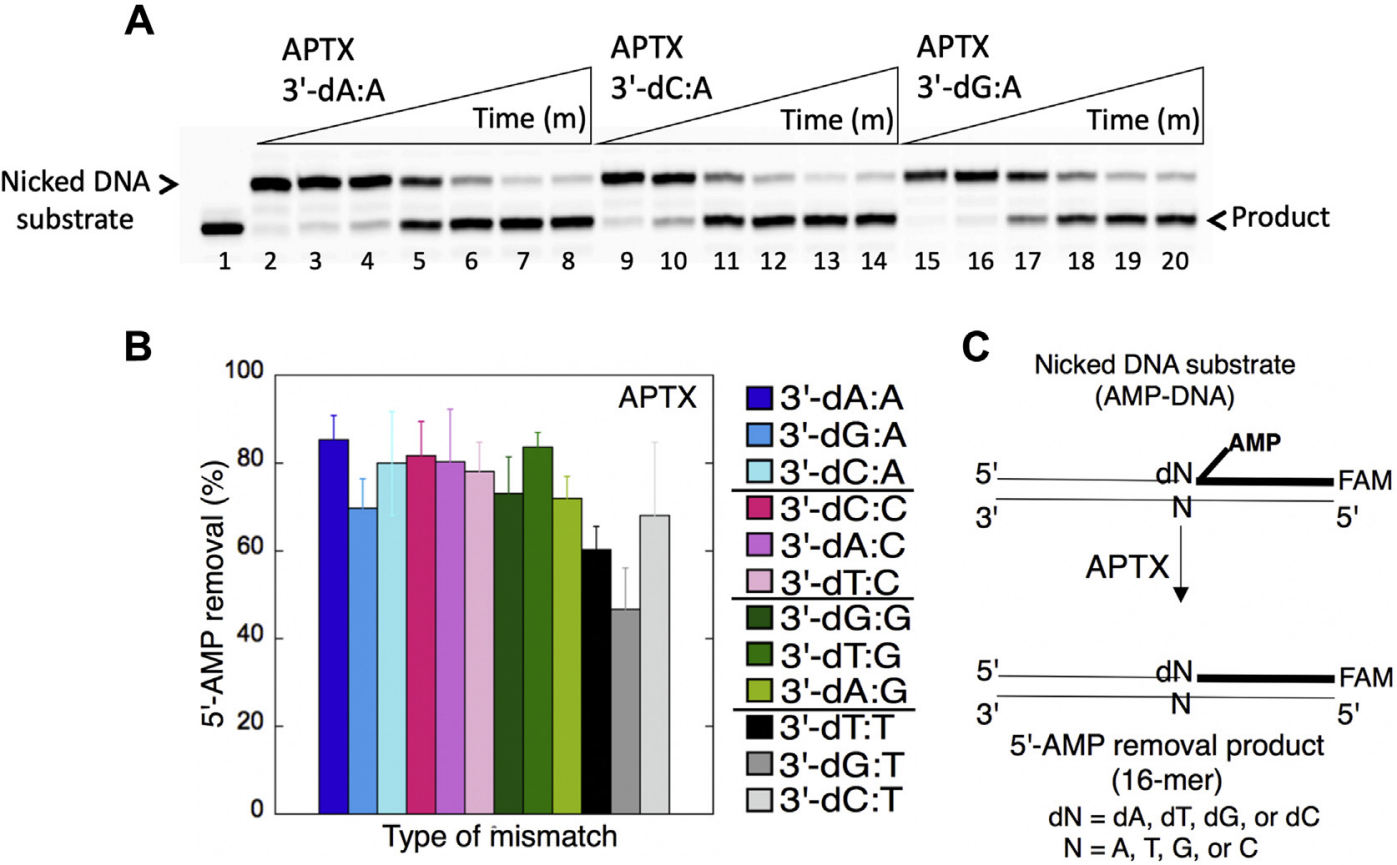
**Removal of 5'-AMP by APTX from the ligation failure products with preinserted 3'-mismatches.***A*, lane 1 is a size marker that corresponds to the oligonucleotide without an AMP moiety, and lane 2 is the negative enzyme control of the nicked DNA substrate with 5'-AMP and 3'-mismatched base. Lanes 3 to 8, 9 to 14, and 15 to 20 show the products of 5'-AMP removal from 3'-dA:A, 3'-dC:A, and 3'-dG:A mismatch-containing substrates, respectively, obtained at the time points 0.5, 1, 3, 5, 8, and 10 min. *B*, the graph shows the comparisons in the products of 5'-AMP removal between all 12 noncanonical base pair mismatches. The data are presented as the averages from three independent experiments ± SDs. The gel images that show 5'-AMP removal from all other mismatches are presented in Fig. S13. C, illustrations of the nicked DNA substrate with 5'-AMP and 3'-mismatches and the products observed in the APTX reaction. APTX, aprataxin.

**Figure 16:**
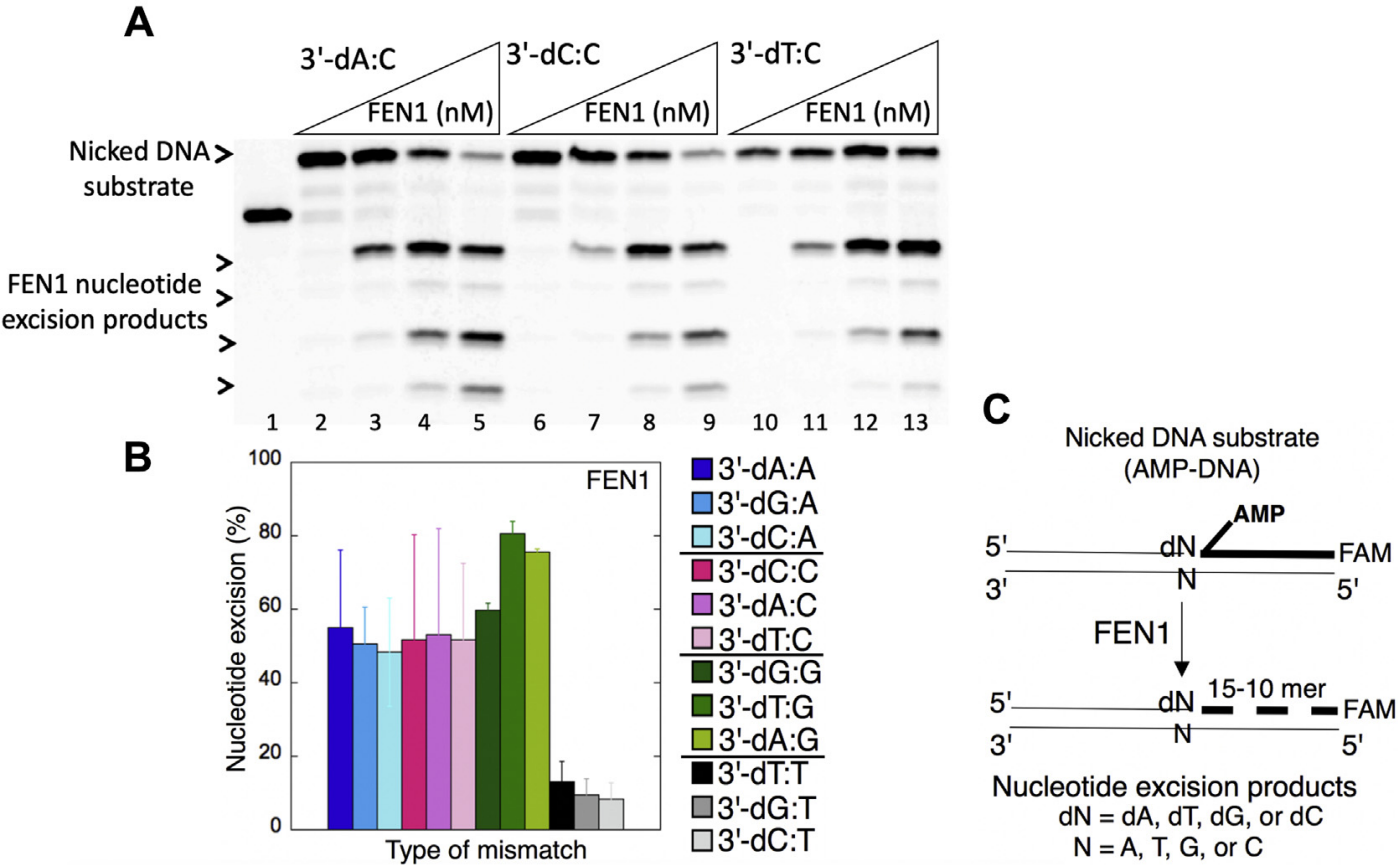
**The nucleotide excisions by FEN1 from the ligation failure products with preinserted 3'-mismatches**. *A*, lane 1 is a size marker that corresponds to the oligonucleotide without an AMP moiety. Lanes 2, 6, and 10 are the negative enzyme controls of the nicked DNA substrates with 5'-AMP and 3'-dA:C, 3'-dC:C, and 3'-dT:C, respectively. Lanes 3 to 5, 7 to 9, and 11 to 13 show the products of nucleotide excisions from 3'-dA:C, 3'-dC:C, and 3'-dT:C mismatch-containing substrates, respectively, obtained as a function of FEN1 concentration. *B*, the graph shows the comparisons in the products of nucleotide excisions for all 12 noncanonical base pair mismatches. The data are presented as the averages from three independent experiments ± SDs. The gel images that show nucleotide excision products from all other mismatches are presented in Fig. S14. *C*, illustrations of the nicked DNA substrate with 5'-AMP and 3'-mismatches and the products observed in the FEN1 reaction. FEN1, flap endonuclease.

We then analyzed APTX and FEN1 activities for the repair intermediates with 5'-AMP and a 3'-preinserted mismatch for all possible 12 noncanonical base pairs (Figs. 15*C* and 16*C*). For example, for the nicked DNA substrates harboring a template base A mismatch, we observed 5'-AMP removal from DNA ends with 3'-preinserted base pairs dA:A, dC:A, and dG:A (Fig. 15*A*, lanes 3–8, 9–14, and 15–20, respectively). Similarly, for the nicked DNA substrates that possess a template base C mismatch, we obtained products of 5'-AMP removal or nucleotide excisions from DNA ends with 3'-preinserted base pairs dA:C, dC:C, and dT:C (Fig. 16*A*, lanes 3–5, 7–9, and 11–13, respectively). Overall, the studies demonstrated 5'-AMP removal by APTX (Fig. S13) and nucleotide excisions by FEN1 (Fig. S14) from all 12 possible mismatch-containing DNA substrates with no significant difference in the specificity for the 3'-mismatch:template base combination (Figs. 15*B* and 16*B*).

## Discussion

LIGs are essential enzymes for DNA replication and repair, and they join DNA strands to finalize each of these processes (1, 2, 3, 4). LIG1 ligates the newly synthesized lagging strand during replication to complete Okazaki fragment maturation and is one of the key enzymes that finalizes the BER pathway after pol β gap filling or the nucleotide insertion step (52). Recently published high-resolution structures of LIG1 in complex with nick DNA have identified the four coordinated Mg^2+^ binding sites including the site 1 at the LIG1–DNA interface that is crucial for the ligase substrate discrimination against damaged DNA ends and reinforces high-fidelity ligation (32). This high-fidelity site, referred as Mg^HiFi^, is not found in the other human LIGs III and IV. The Mg^HiFi^ site is coordinated by two glutamate (E) side chains that reside in the two domains of the LIG1 catalytic core, *that is*, E346 in the DNA-binding domain and E592 in the adenylation domain (Fig. S3). According to the crystal structures of LIG1 bound to adenylated DNA containing 3'-8-oxodG that forms a Hogsten edge to base pair with an adenine base in a template position (PDB: 6P0E), when the high-fidelity residues E592 and E346 are mutated to alanine (EE/AA), the Mg^HiFi^ site moves away from the active site because of the relaxation of the strict geometric requirements for Mg^2+^ metal coordination, which generates an open cavity that accommodates and seals 8-oxodG–containing DNA termini (32). The structural comparisons of the LIG1 EE/AA in complex with the nick DNA including 8-oxodG:A *versus* WT LIG1 with undamaged nicked DNA complex including dG:C demonstrated the significant structural adjustments such as loss of bound metal ions, conformational displacements of bound nicked DNA at upstream strand, and displacements at LIG1–DNA van der Waals contacts, which all contribute to an essential role of Mg^HiFi^ for enforcing the 3'-strand end recognition (32).

In the present study, we comprehensively characterized the importance of LIG1 Mg^HiFi^ site in terms of BER regulation and the ligase substrate discrimination *in vitro*. For the first time, we studied the ligation efficiency of pol β–oxidized or mismatch nucleotide insertion products by LIG1 low-fidelity mutants, which mimic the substrate–product channeling between pol β and LIG1 when the fidelity of last nick-sealing step in the coordinated BER pathway is perturbed. Our results revealed that the LIG1 EE/AA is capable of mutagenic ligation of pol β 8-oxodGTP insertion products. We have termed mutagenic ligation because the LIG1 low-fidelity mutant was able to accommodate an 8-oxodGMP inserted by pol β and seal the nicked insertion product including this oxidatively damaged base lesion that is a commonly found base modification in mammalian DNA and is known to be mutagenic *in vitro* and *in vivo* (41). Interestingly, the mutagenic ligation after pol β 8-oxodGTP insertion opposite A is much more efficient and faster than that of 8-oxodGTP opposite C. This could be due to the preferential insertion characteristic of pol β for 8-oxodG favoring synconformation that can form a Hoogsteen base pair with adenine (45, 46).

On the other hand, according to our coupled-reaction results for the ligation of pol β mismatch insertion, the low-fidelity LIG1 carries out the ligation (which we have termed self-ligation) of the one-nucleotide-gap DNA substrate itself. This self-ligation results in a ligation product one nucleotide shorter than the predicted completed ligation of the nicked pol β insertion product. This characteristic of the low-fidelity ligase was also validated in the ligation reactions with an increased affinity of the EE/AA mutant to bind and ligate the gap over nick DNA. Our results suggest that the mutations at Mg^HiFi^ sites did not reduce catalytic activity for sealing of nicked insertion products with correctly base-paired ends (*i.e.*, dA:T), whereas the EE/AA mutation induces direct ligation of a gap DNA in case pol β inserts a mismatch with lower efficiency (*i.e.*, dG:T) into this gap repair intermediate. This suggests that DNA polymerase–mediated aberrant or inefficient nucleotide insertions could lead to the formation of mutagenic repair or replication intermediates in case of perturbed LIG1 fidelity during downstream steps of BER or Okazaki fragment maturation on the lagging strand. Recently, the potential importance of high-fidelity ligation during Okazaki fragment maturation of DNA replication has been reported (53). Moreover, previous studies have described unusual conformations of pol β bound to mismatched substrates, including G-T mismatches in a wobble conformation (54, 55, 56, 57, 58). Furthermore, the mismatches having transient Watson–Crick geometry at the polymerase active site have been considered as a source of spontaneous mutagenesis and base substitution errors during DNA replication (47, 48, 49, 50, 51). We suggest that the EE/AA LIG1 could also exhibit such noncatalytic conformation with an active site geometry that cannot accommodate a Watson–Crick–like G-T base pairing.

Our overall findings of the coupled assay system suggest the model where the coordination between pol β and LIG1 at the downstream steps of BER would be important for controlling the channeling of toxic and mutagenic DNA repair intermediates, and the mutations that govern the LIG1 fidelity could affect repair outcomes (Scheme S1). These elements could be biologically relevant in cellular conditions or where genetic mutations reduce fidelity of pol β–mediated and/or LIG1-mediated DNA repair, thereby contributing to the development of neurological diseases or cancer. For example, studies suggest a pivotal role for pol β–mediated high-fidelity nucleotide insertion during the gap-filling step of BER in the prevention of carcinogenesis (59). Moreover, mutations identified in the LIG1 gene of human patients who exhibit the symptoms of developmental abnormalities, immunodeficiency, and lymphoma indicate that LIG1 defects cause genetic instability and predisposition to cancer (60, 61, 62). Our study demonstrates how the possible mutagenic consequences after pol β misinsertion is mediated by LIG1. Because pol β, the main BER polymerase with no proofreading activity, has a relatively low inherent fidelity, LIG1 fidelity is vital for preserving faithful BER, especially under oxidative stress conditions in cells.

In addition to our findings from coupled BER reactions that included pol β and LIG1, in the present study, we also comprehensively analyzed the role of the Mg^HiFi^ site on the substrate specificity of LIG1 against the nicked repair intermediates with preinserted 3'-damaged or mismatched ends that mimic pol β–oxidized or mismatch insertion products. Our results revealed ligation failure of repair intermediates containing preinserted 3'-8-oxodG when paired with template G or T in contrast to the mutagenic ligation of DNA ends with 3'-8-oxodG opposite A or C by the LIG1 EE/AA mutant. This was also the case for WT LIG1. The more efficient ligation of nicked DNA with preinserted 3'-8-oxodG opposite A over C by LIG1 EE/AA is consistent with the enhanced mutagenic ligation products after pol β 8-oxodGTP insertion opposite A over C. This difference we observed similarly in both cases could be due to the dual coding potentials of 8-oxodG (anti or syn). We suggest that the LIG1 active site could exhibit distinct structural conformations to accommodate the primer terminus with an 8-oxodGMP possibly stemming from the dual coding potentials of 8-oxodG base (anticonformation *versus* synconformation).

Unfortunately, there is only one LIG1 structure solved for the damaged DNA complex with 8-oxodG opposite A to be able to interpret our other base-pairing findings mechanistically. We suggest that the existence of G or T in the template DNA strand does not align the catalytic participants for optimal chemistry, preventing ligation of the damaged termini at the LIG1 active site to accommodate template base G or T while engaging the primer terminus with a damaged base to be ligated. We suggest that the active-site geometry of LIG1 could serve as an additional fidelity checkpoint for DNA ends containing 3'-8-oxodG opposite G or T, where the intrinsic poor geometry deters mutagenic ligation despite mutations at the Mg^HiFi^ site. Similarly, the crystal structures of Y-family DNA polymerase iota demonstrated poor hydrogen-bonding properties and unfavorable conformations for dTTP and dGTP nucleotides for replication opposite 8-oxodG (44).

In the present study, we also demonstrated distinct nick-sealing ability of DNA ends with noncanonical base pairs by the LIG1 low-fidelity mutants. Our structural homology models of LIG1 for the WT and EE/AA show the changes in the nucleotide residue contacts at the active site in complex with the nicked DNA, which demonstrates the formation of an open cavity along with a displacement of the Mg^HiFi^ site that could significantly affect the accommodation of 3'-end base-paired bases at the upstream strand (Fig. S15). According to our models, the mutagenesis at both conserved glutamate residues (E346 and E592) is required for the formation of the cavity as the Mg^HiFi^ metal site can still confer high fidelity in case of single amino acid mutations (Fig. S16). This could explain the differences we observed in the ligation efficiency of 3'-mismatched DNA ends between 2 single (E346A and E592A) and 1 double (EE/AA) low-fidelity mutants of LIG1.

For LIGs from other sources, the study with *Tth* ligase from *Thermus thermophilus HB8* revealed that the base pair geometry is much more important than relative base pair stability and that the ligase probes the hydrogen bond acceptors present in the minor groove to ensure fidelity (63). Similarly, the importance of base pair geometry for the fidelity of DNA synthesis has been suggested for repair and replication DNA polymerases (54, 55, 64, 65). We suggest that LIG1 could use different conformational checkpoints to hinder sealing noncanonical ends and this depends on the composition of mismatched base pairs at the active site as reported by structural studies of DNA polymerases (65). Although it appears to be mechanistically similar to the incorporation of deoxynucleotide triphosphates by DNA polymerases, further structural studies are required to understand the molecular process by which human LIG1 fidelity is accomplished to gain insight into the mechanistic basis for discrimination against the range of substrates that can harbor aberrant base pair architecture.

As persistent DNA breaks are expected to block transcription or be converted into double-strand breaks during DNA replication, the formation and repair of ligation failure intermediates are expected to be critical to cell viability and genome stability (9, 10). In our previous studies, we reported the formation of 5'-AMP–containing BER intermediates after pol β 8-oxodGTP insertion *in vitro* and an increased cytotoxicity to an oxidative stress–inducing agent in pol β WT cells relative to pol β–null cells *in vivo* (13). The accumulation of adenylate intermediates in cells could be toxic, leading to DNA strand break products with 8-oxodG or AMP lesions. In the present study, for the first time, we comprehensively characterized the substrate specificity of APTX for the mutagenic ligation failure intermediates with 5'-AMP and 3'-oxidative damage base or noncanonical base pairs. We demonstrated that APTX, as a DNA-end processing protein, is able to effectively remove the 5'-AMP block from these repair intermediates that mimic pol β mismatch or oxidized base insertion products. The mutations in the APTX gene (*aptx*) are linked to the autosomal recessive neurodegenerative disorder AOA1 (33, 37). APTX-null cells fail to show a hypersensitivity phenotype to DNA damage–inducing agents, and normal repair of base lesions and strand breaks have been reported in an APTX-deficient mouse model (66, 67). We previously reported the complementary role of FEN1 in cases of deficiency in APTX enzymatic activity in cell extracts from patients with AOA1 (34, 35, 36). In the present study, our findings also indicate the presence of a compensatory role of FEN1 in case of APTX deficiency to deadenylate ligation failure products that can be formed *via* pol β–promoted mutagenic nucleotide insertions and aberrant LIG1 fidelity. These are important findings in this context because we showed WT LIG1 is much more prone to failing and producing abortive ligation products than the low-fidelity mutant. Conversely, to enable repair to proceed, an additional 3'-end processing by a proofreading enzyme, such as APE1 or mismatch repair protein (*i.e.*, MSH2), is required for the removal of the damaged or mismatched base (68, 69, 70).

Further structural and biochemical studies are required to understand how the repair enzymes (pol β, APE1, LIG1, APTX, and FEN1) function together in a multiprotein/DNA complex to facilitate the faithful channeling of DNA repair intermediates. Moreover, to elucidate the potential contribution of crosstalk between these BER players and mismatch repair proteins, as well as several proteins related to the DNA damage response (*i.e.*, XRCC1 which interacts with pol β, APE1, and APTX), cell studies should be considered to gain further insight into the determinants of repair fidelity. In the future, it may be possible to develop small molecule inhibitors against the protein-interacting partners of human LIG1 that could potentiate the effects of chemotherapeutic compounds and improve cancer treatment outcomes (9).

## Experimental procedures

### Preparation of DNA substrates

Oligodeoxyribonucleotides with and without a 6-carboxyfluorescein label and the 5'-adenylate (AMP) were obtained from Integrated DNA Technologies. The DNA substrates used in this study were prepared as described previously (12, 13, 14, 15, 16, 34, 35, 36, 71, 72). The one-nucleotide-gap DNA substrates were used for coupled assays (Table S1). The nicked DNA substrates containing template base A, T, G, or C and 3'-preinserted bases (dA, dT, dG, or dC) or 3'-8-oxodG were used for DNA ligation assays (Table S2). The nicked DNA substrates containing template base A, T, G, or C as well as 5'-AMP and 3'-preinserted bases (dA, dT, dG, or dC) or 3'-8-oxodG were used for APTX and FEN1 activity assays (Table S3).

### Protein purifications

The constructs for LIG1 mutants (E346A, E592A, and E346A/E592A) were prepared using the WT full-length LIG1 (pET-24b) and site-directed mutagenesis with synthetic primers and confirmed by sequencing of the coding region. The His-tag recombinant for LIG1 low-fidelity mutants were purified as described previously for WT LIG1 with slight modifications (12, 13, 14, 15, 16). Briefly, the protein was overexpressed in Rosetta (DE3) pLysS *E. coli* cells (Millipore Sigma) and grown in Terrific Broth media with kanamycin (50 μg ml^−1^) and chloramphenicol (34 μg ml^−1^) at 37 °C. Once the absorbance reached 1.0, the cells were induced with 0.5-mM IPTG and the overexpression was continued overnight at 16 °C. After the centrifugation, the cell was lysed in the lysis buffer (50-mM Tris HCl, pH 7.0, 500-mM NaCl, 20-mM imidazole, 10% glycerol, 1-mM PMSF, an EDTA-free protease inhibitor cocktail tablet) by sonication at 4 °C. The lysate was pelleted at 16,000*g* rpm for 1 h at 4 °C. The cell lysis solution was filter-clarified and then loaded onto a HisTrap HP column (GE Health Sciences) that was previously equilibrated with binding buffer A (50-mM Tris HCl, pH 7.0, 500-mM NaCl, 20-mM imidazole, 10% glycerol). The column was washed with binding buffer A and then followed by buffer B (50-mM Tris HCl, pH 7.0, 500-mM NaCl, 35-mM imidazole, 10% glycerol). The protein was eluted with an increasing imidazole gradient (0–500 mM) of the elution buffer A at 4 °C. The collected fractions were then subsequently loaded onto HiTrap Heparin (GE Health Sciences) column that was equilibrated with binding buffer C (50-mM Tris HCl, pH 7.0, 50-mM NaCl, and 10% glycerol), and protein is eluted with elution buffer D (20-mM Tris HCl, pH 7.0, 1 M NaCl, and 10% glycerol). The LIG1 protein was further purified by Resource Q and finally by Superdex 200 10/300 (GE Health Sciences) columns in the buffer (20-mM Tris HCl, pH 7.0, 200-mM NaCl, 2-mM β-mercaptoethanol, and 5% glycerol).

Recombinant (GST-tagged pGEX-4T-3) WT full-length human DNA polymerase (pol) β was purified as described previously (12, 13, 14, 15, 16). Briefly, the protein was overexpressed in One Shot BL21(DE3)pLysS *E. coli* cells (Invitrogen) and grown at 37 °C with 0.5-mM IPTG induction. The cells were then grown overnight at 16 °C. After centrifugation, the cells were lysed at 4 °C by sonication in lysis buffer containing 1× PBS (pH 7.3) and 200-mM NaCl, and a protease inhibitor cocktail. The lysate was pelleted at 16,000*g* rpm for 1 h and then clarified by filtration. The pol β supernatant was loaded onto a GSTrap HP column (GE Health Sciences) that was equilibrated with 1× PBS (pH 7.3) and purified with the elution buffer containing 50-mM Tris HCl (pH 8.0), 10-mM reduced glutathione, and 1-mM DTT at 4 °C. The collected fractions were subsequently passed through a HiTrap Desalting HP column in a buffer containing 150-mM NaCl and 20-mM NaH_2_PO4 (pH 7.0), and then further purified by Superdex 200 Increase 10/300 chromatography (GE Healthcare). All proteins purified in this study were dialyzed against storage buffer (25-mM Tris HCl, pH 7.4, 100-mM KCl, 1-mM TCEP, and 10% glycerol), concentrated, frozen in liquid nitrogen, and stored at −80 °C. Protein quality was evaluated onto 10% SDS-PAGE, and the protein concentration was measured using absorbance at 280 nm.

### Coupled assays

The coupled assays were performed to measure pol β and LIG1 activities simultaneously in the same reaction mixture as described previously (12, 13, 14, 15, 16, 71, 72). For this purpose, we used the one-nucleotide-gap DNA substrates (Table S1). Briefly, the reaction was initiated by the addition of preincubated enzyme mixture including pol β (10 nM) and LIG1 (10 nM) to a reaction mixture containing 50-mM Tris HCl (pH 7.5), 100-mM KCl, 10-mM MgCl_2_, 1-mM ATP, 1-mM DTT, 100 μg ml^−1^ bovine serum albumin (BSA), 10% glycerol, the DNA substrate (500 nM), and 8-oxodGTP or dGTP (100 μM). The reaction mixture was incubated at 37 °C and stopped at the indicated time points in figure legends. The reaction products were quenched with the addition of the gel loading buffer (95% formamide, 20-mM ethylenediaminetetraacetic acid, 0.02% bromophenol blue, and 0.02% xylene cyanol) and then separated by electrophoresis on an 18% polyacrylamide gel. The gels were scanned with a Typhoon PhosphorImager (Amersham Typhoon RGB), and the data were analyzed using ImageQuant software. The coupled assays were performed similarly for WT and low-fidelity LIG1 mutants E346A, E592A, or EE/AA.

### DNA-ligation assays

The ligation assays were performed to analyze the substrate specificity of LIG1 as described previously (12, 13, 14, 15, 16, 71, 72). For this purpose, we used the nicked DNA substrates with 3'-preinserted 8-oxodG or mismatched bases (Table S2). Briefly, the reaction was performed in a mixture containing 50-mM Tris HCl (pH 7.5), 100-mM KCl, 10-mM MgCl_2_, 1-mM ATP, 1-mM DTT, 100 μg ml^−1^ BSA, 10% glycerol, the nicked DNA substrate (500 nM), and LIG1 (100 nM). The reaction mixtures were then incubated at 37 °C for the times indicated in figure legends and were quenched by mixing with an equal volume of the loading dye. The products were separated, and the data were analyzed as described above. The ligation assays were performed similarly for WT and low-fidelity LIG1 mutants E346A, E592A, or EE/AA.

### APTX and FEN1 assays

APTX and FEN1 activity analyses were performed as described previously (34, 35, 36). For this purpose, we used the nicked DNA substrates containing 5'-AMP and 3'-preinserted damaged (8-oxodG) or all possible 12 mismatched bases (Table S3). Briefly, the APTX assay was performed in a mixture containing 50-mM Tris HCl (pH 7.5), 40-mM KCl, 5-mM EDTA, 1-mM DTT, 5% glycerol, and the nicked DNA substrate (500 nM). The FEN1 assay was performed in a mixture containing 50-mM Hepes (pH 7.5), 20-mM KCl, 0.5-mM EDTA, 2-mM DTT, 10-mM MgCl_2_, and the nicked DNA substrate (500 nM). For both assays, the reactions were initiated with the addition of APTX (100 nM) or FEN1 (100–500 nM) and incubated at 37 °C. The activity assays were stopped at 15 min for FEN1 and at the indicated time points in figure legends for APTX by mixing with an equal volume of the loading dye. The reaction products were then analyzed as described above.

### Structure modeling

Structure analysis was performed based on the crystal structure of LIG1 (PDB: 6P0E) using the Coot software (73). All structural images were drawn using PyMOL (http://www.pymol.org/).

### BLI assays for LIG1–DNA interactions

The real-time monitoring of binding kinetics for protein–DNA interactions of LIG1 (WT and EE/AA mutant) were performed using the BLI assay by the Octet QKe system (ForteBio). The oligonucleotide with 3'-Biotin label was obtained from Integrated DNA Technologies. The one-nucleotide-gap DNA substrate with template T was prepared as described above (Table S4). Streptavidin biosensors (ForteBio) were used to attach the biotin-labeled DNA. The BLI experiments were performed at 20 °C in 96-well microplates with agitation set to 1000 rpm. The streptavidin biosensors were hydrated in the kinetics buffer (Hepes, pH 7.4, 200-mM NaCl, 0.5% BSA, 0.05% Tween 20) at 20 ºC for 20 min. The sensors were then immersed in DNA (40 nM) in the kinetics buffer for 300 s. After recording an initial baseline in the kinetics buffer (60 s), the sensors with DNA were exposed to the concentration range of LIG1 protein (10–2430 nM) for 240 s association and then in kinetics buffer for 240 s dissociation. The association (k_on_) and dissociation (k_off_) rates and the binding affinity were calculated using the ForteBio Data Analysis software with 1:1 binding model.

## Data availability

All data are contained within the article. Further information and requests of materials used in this research should be directed to Melike Çaglayan (caglayanm@ufl.edu). Plasmid DNA constructs generated in this study will be made available *via* material transfer agreement (MTA).

## Supporting information

This article contains supporting information.

## Conflict of interest

The authors declare that they have no conflicts of interest with the contents of this article.

## Supporting information

Supplemental Figures S1–S16, Scheme S1 and Tables S1–S4
