# Supplementary material for: DNA ligase I fidelity mediates the mutagenic ligation of pol β oxidized and mismatch nucleotide insertion products in base excision repair
Source: J Biol Chem. 2021 Feb 16;296:100427. doi: 10.1016/j.jbc.2021.100427 (PMC8024709; doi:10.1016/j.jbc.2021.100427)
Supplement: Supplemental Figures S1–S16, Scheme S1 and Tables S1–S4 [file mmc1.pdf]

DNA ligase I fidelity mediates the mutagenic ligation of pol β oxidized and mismatch nucleotide insertion products in base excision repair

Pradnya Kamble, Kalen Hall, Mahesh Chandak, Qun Tang, Melike Çağlayan

Department of Biochemistry and Molecular Biology, University of Florida, Gainesville, FL 32610, USA

To whom correspondence should be addressed. Tel.: +1 352-294-8383; Email: caglayanm@ufl.edu

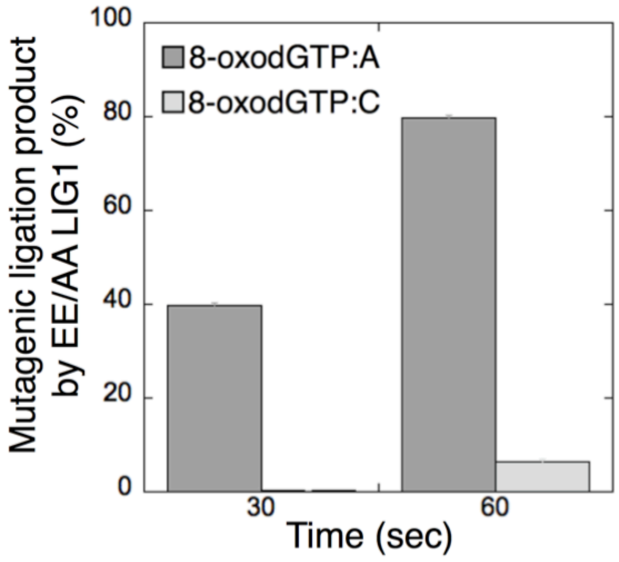

Figure S1. The comparison of mutagenic ligation products after pol β 8-oxodGTP insertions opposite A vs C by LIG1 E346A/E592A mutant. The data as the averages from three independent experiments ± SDs are presented in Figs. 1 and 2.

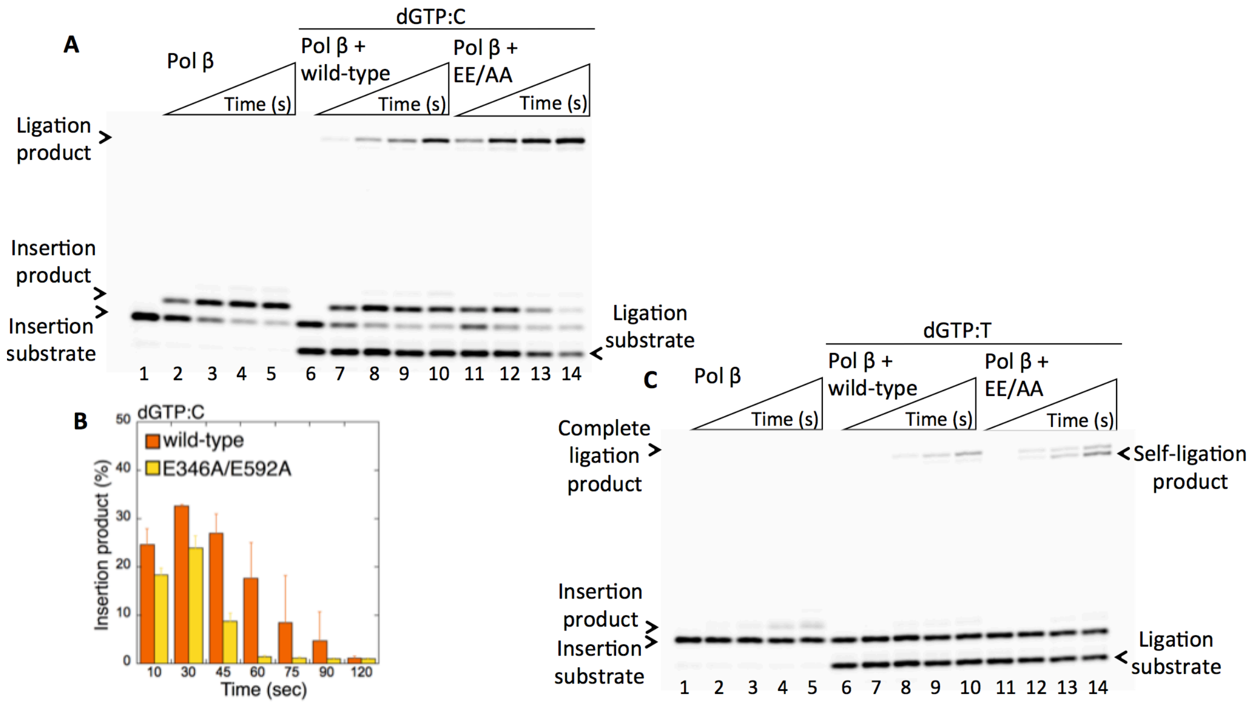

Figure S2. Pol β dGTP:C vs dGTP:T insertions and ligation of these insertion products by LIG1 wild-type and E346A/E592A (EE/AA) mutant. *A*, lanes 1 and 6 are the negative enzyme controls of the one nucleotide gap DNA substrates with template base C for the nucleotide insertion and coupled reactions, respectively. Lanes 2-5 show the pol β dGTP:C insertion products obtained at the time points 10s, 30s, 45s, and 60s. Lanes 7-10 and 11-14 are the ligation products after pol β dGTP:C insertion by LIG1 wild-type and EE/AA mutant, respectively, obtained at the time points 10s, 30s, 45s, and 60s. *B*, the graph show the time-dependent changes in the pol β dGTP:C insertion products. The data as the averages from three independent experiments ± SDs are presented in Fig. 3. *C*, lanes 1 and 6 are the negative enzyme controls of the one nucleotide gap DNA substrates with template base T for the nucleotide insertion and coupled reactions, respectively. Lanes 2-5 show the pol β dGTP:T insertion products obtained at the time points 10s, 30s, 45s, and 60s. Lanes 7-10 and 11-14 are the ligation products after pol β dGTP:T insertion by LIG1 wild-type and EE/AA mutant, respectively, obtained at the time points 10s, 30s, 45s, and 60s.

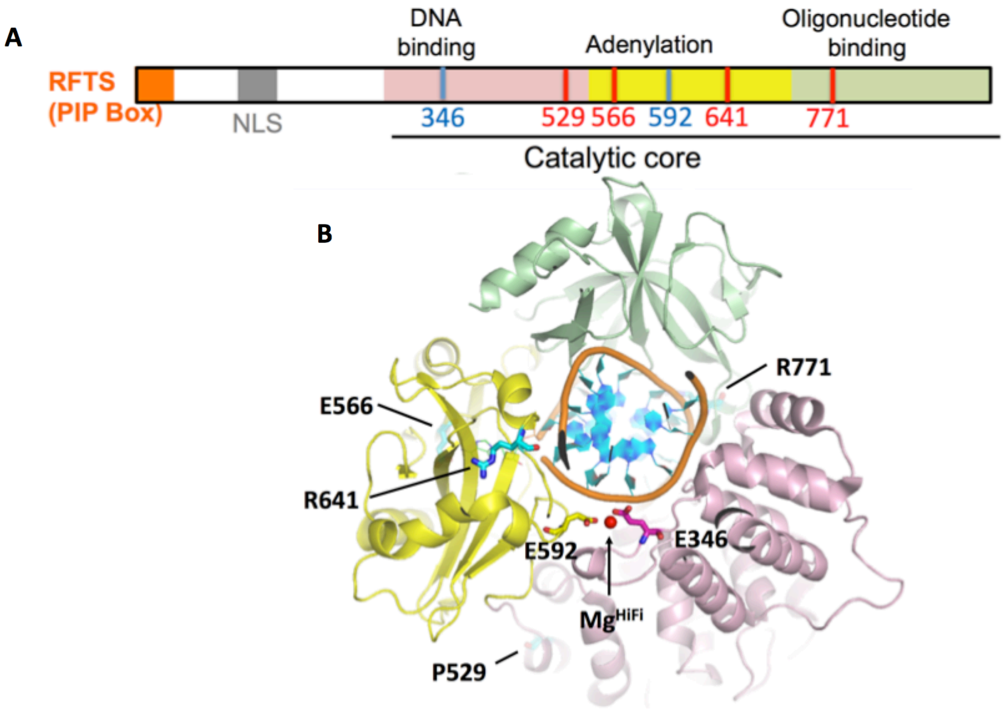

Figure S3. Domain architecture of LIG1 with the low fidelity mutants and LIG deficiency disease-associated mutations. *A*, schematic view showing the domain composition of human LIG1 including the N-terminal domain (gray), and the catalytic core consisting of the DNA-binding domain (pink), Adenylation domain (yellow), and oligonucleotide binding domain (green). *B*, LIG1 (cartoon) in complex with an adenylylated nicked DNA complex (stick, orange). The amino acid residues (P529, E566, R641, and R771) that are mutated in LIG1-deficiency disease and the amino acid residues (E346 and E592) that reinforce the ligase fidelity are shown as sticks.

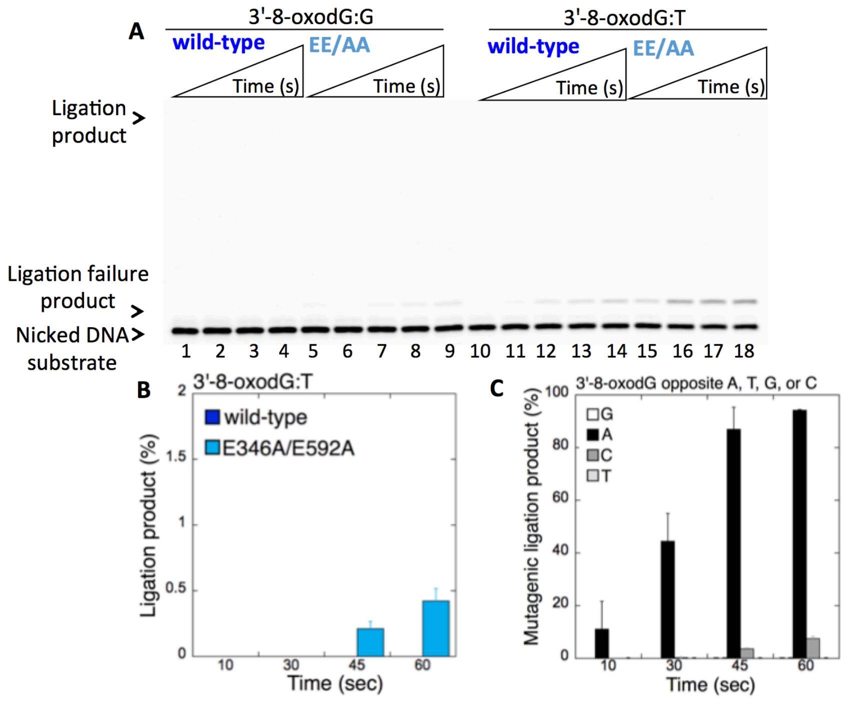

Figure S4. Ligation efficiency of the repair intermediate with 3'-8-oxodG opposite G or T by LIG1 wild-type and low fidelity E346A/E592A (EE/AA) mutant. *A*, lanes 1 and 10 are the negative enzyme controls of the nicked DNA substrate with 3'-8-oxodG opposite template base G and T, respectively. Lanes 2-5 and 6-9 show the ligation products of the nicked DNA substrate with 3'-8-oxodG:G by LIG1 wild-type and EE/AA mutant, respectively, obtained at the time points 10s, 30s, 45s, and 60s. Lanes 11-14 and 15-18 show the ligation products of the nicked DNA substrate with 3'-8-oxodG:T by LIG1 wild-type and EE/AA mutant, respectively, obtained at the time points 10s, 30s, 45s, and 60s. *B*, the graph shows the time-dependent changes in the ligation products for the 3'-8-oxodG:T nicked DNA and the data are presented as the averages from three independent experiments ± SDs. *C*, the comparison of mutagenic ligation products for the nicked DNA with 3'-8-oxodG opposite all four template bases by LIG1 EE/AA mutant. The data as the averages from three independent experiments ± SDs are presented in this figure (*B*) and Fig. 5 (*C*).

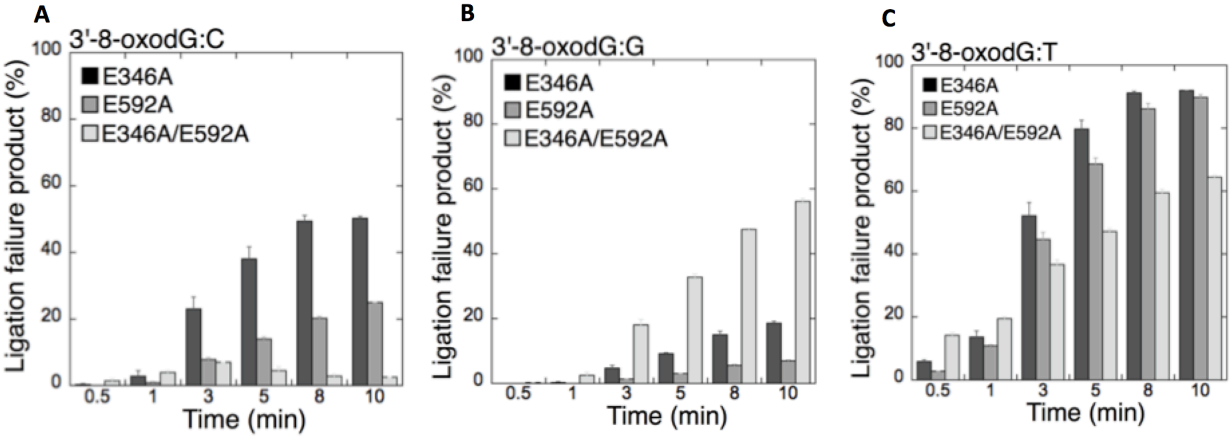

Figure S5. The comparison of ligation failure products for the nicked DNA substrates including preinserted 3'-8-oxodG by LIG1 low fidelity mutants. *A-C*, the data as the averages from three independent experiments ± SDs are presented in Fig. 6 (*A*) and Fig. 7 (*B,C*).

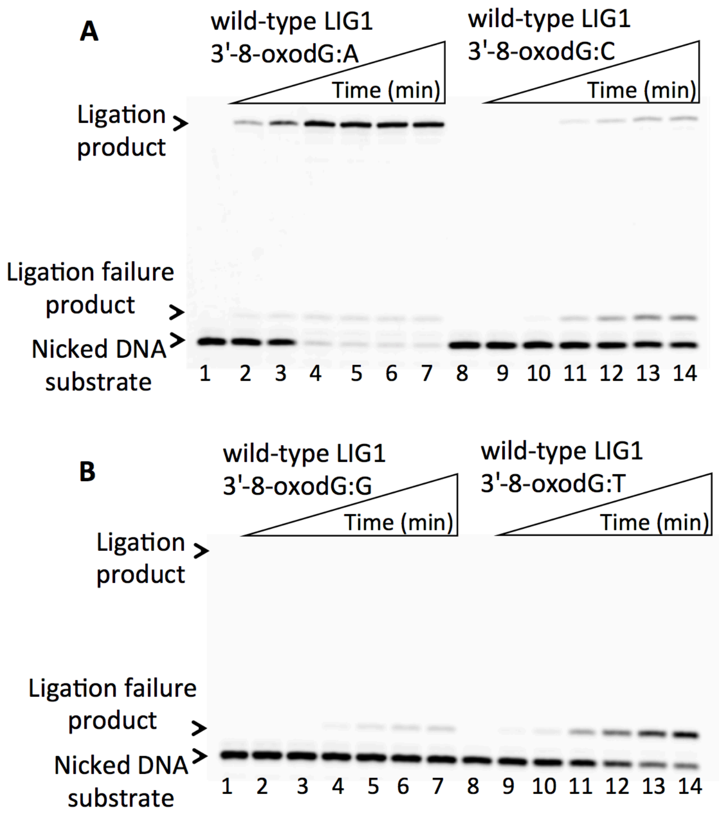

Figure S6. Ligation of the repair intermediate with preinserted 3'-8-oxodG by LIG1 wild-type. *A*, lanes 1 and 8 are the negative enzyme controls of the nicked DNA substrates with 3'-8-oxodG opposite template base A or C, respectively. Lanes 2-7 and 9-14 show the ligation products for 3'-8-oxodG:A and 3'-8-oxodG:C substrates, respectively, obtained at the time points 0.5, 1, 3, 5, 8, and 10 min. *B*, lanes 1 and 8 are the negative enzyme controls of the nicked DNA substrates with 3'-8-oxodG opposite template base G or T, respectively. Lanes 2-7 and 9-14 show the ligation products for 3'-8-oxodG:G and 3'-8-oxodG:T substrates, respectively, obtained at the time points 0.5, 1, 3, 5, 8 and 10 min.

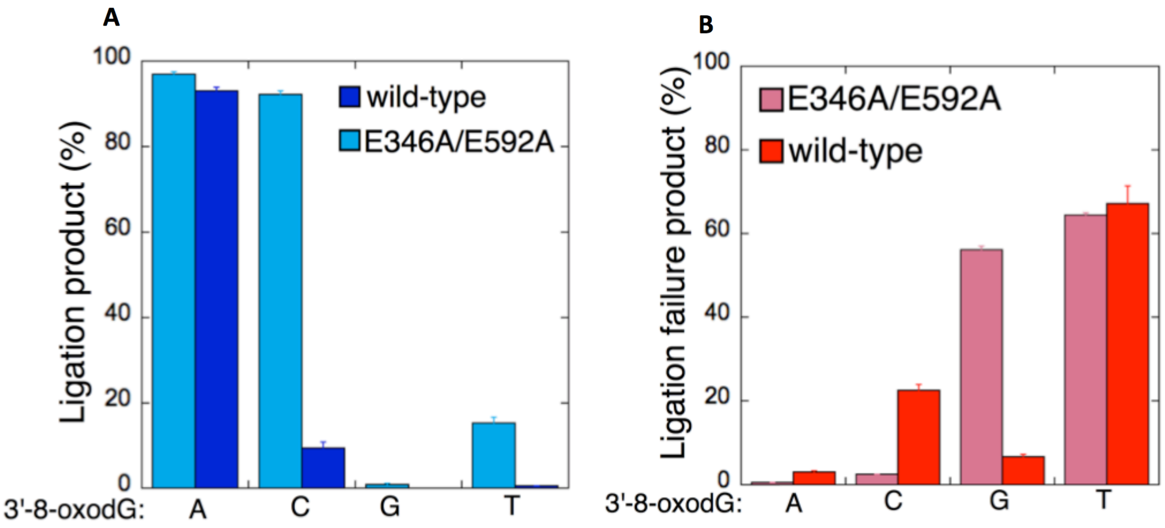

Figure S7. The comparisons for the ligation efficiency of the repair intermediates with preinserted 3'-8-oxodG by LIG1 wild type and low fidelity EE/AA mutant. The graphs show the time-dependent changes in the ligation (*A*) and ligation failure (*B*) products. The data (for 10 min time point) are presented as the averages from three independent experiments ± SDs. The results gel images are presented in Figs. 6 and 7.

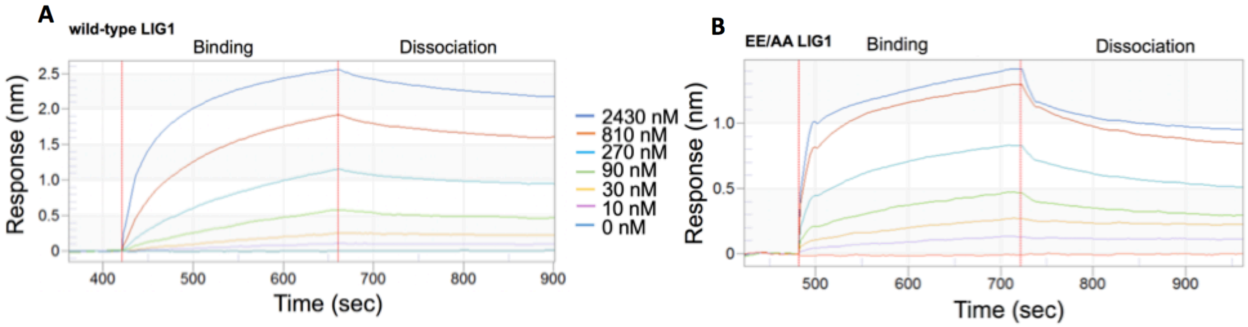

C

	wild-type LIG1	EE/AA LIG1
K_D (nM)	83	18.2
k_{on} (1/M x s)	1.08×10^4	9.31×10^4
k_{off} (1/s)	8.94×10^{-4}	1.7×10^{-3}

Figure S8. The effect of EE/AA low fidelity mutation on the one nucleotide gap DNA binding affinity of LIG1. Table (*C*) shows the values for the association (k_{on}), dissociation (k_{off}) and binding (K_D) values for LIG1 wild type (*A*) vs EE/AA mutant (*B*).

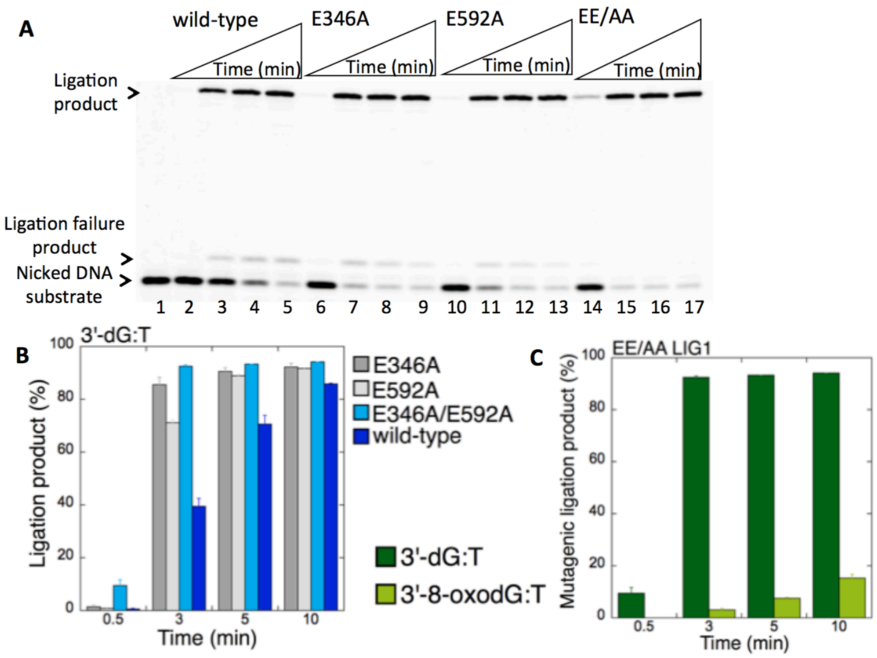

Figure S9. Ligation efficiency of the repair intermediate with Watson-Crick-like 3'-dG:T mismatch by low fidelity LIG1 mutants. *A*, lane 1 is the negative enzyme control of the nicked DNA substrate with 3'-dG:T mismatch, and lanes 2-5, 6-9, 10-13, and 14-17 show the ligation products by LIG1 wild type, E346A, E592A, and E346A/E592A mutants, respectively, obtained at the time points 0.5, 3, 5, 10 min. *B*, the graph shows the time-dependent changes in the ligation products and the data are presented as the averages from three independent experiments ± SDs. *C*, the comparison of mutagenic ligation products for the nicked DNA substrates including preinserted 3'-8-oxodG:T vs 3'-dG:T by LIG1 EE/AA mutant. The data as the averages from three independent experiments ± SDs are presented in this figure and Fig. 7*C-D*.

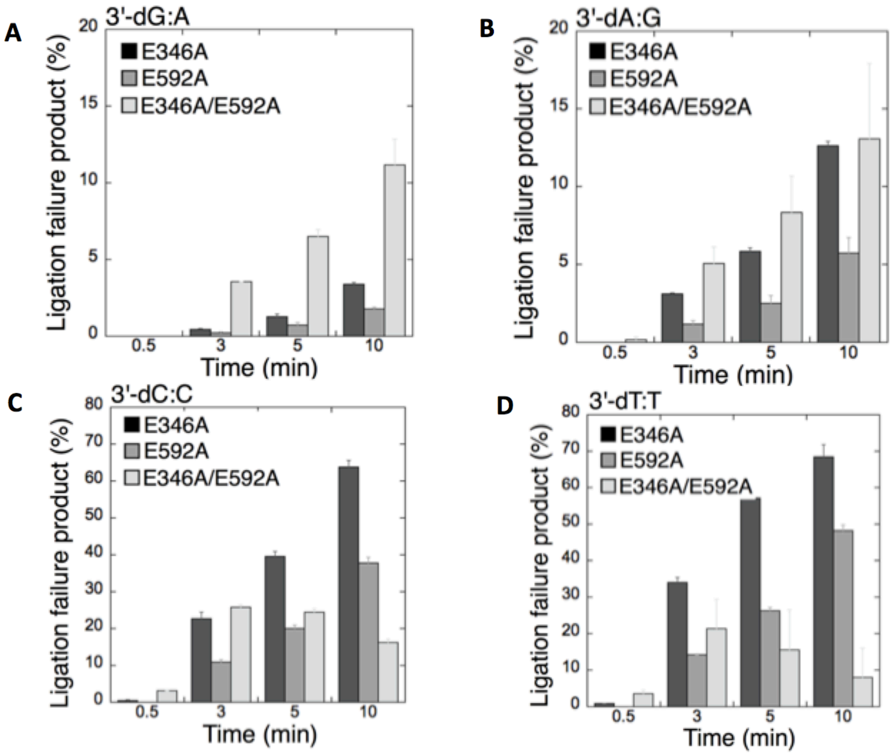

Figure S10. The comparisons for the ligation efficiency of the repair intermediates with preinserted 3'-mismatched bases by LIG1 low fidelity mutants. The graphs show the time-dependent changes in the ligation failure products and the data are presented as the averages from three independent experiments ± SDs. The results gel images are presented in Figs. 11 and 12.

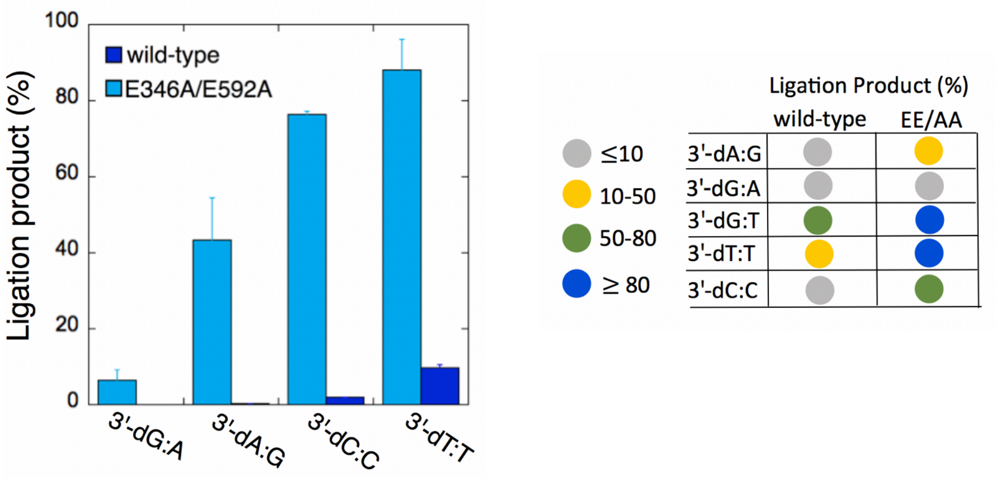

Figure S11. The comparisons for the ligation efficiency of the repair intermediates with preinserted 3'-mismatched by LIG1 wild type and low fidelity EE/AA mutant. The graph shows the time-dependent changes in the ligation products. The data (for 10 min time point) are presented as the averages from three independent experiments ± SDs. The results gel images are presented in Figs. 11 and 12.

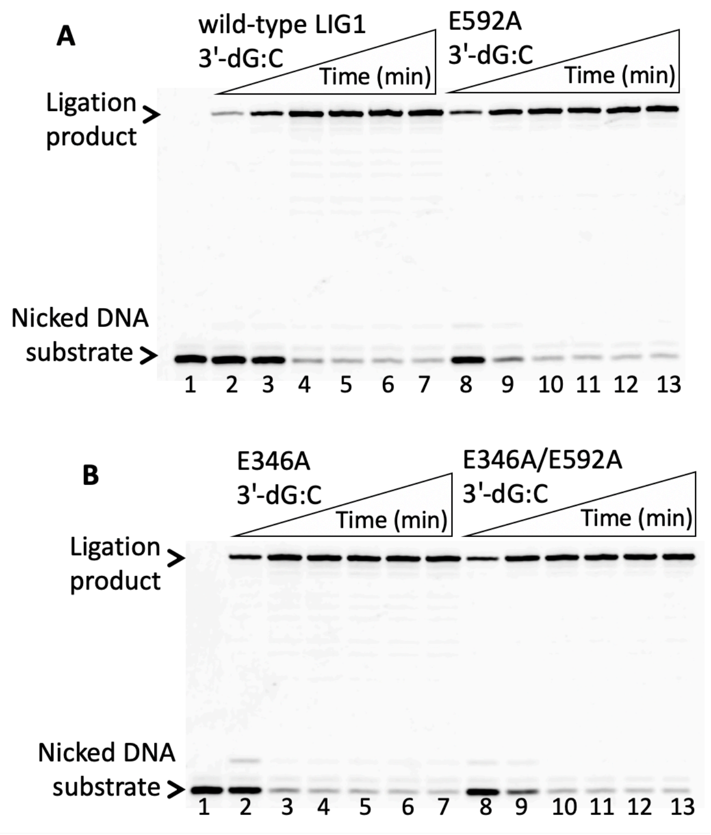

Figure S12. Ligation of the repair intermediate with preinserted 3'-dG:C by LIG1 wild type and low fidelity mutant E346A/E592A. *A*, lane 1 is the negative enzyme control of the nicked DNA substrate with 3'-dG opposite template base C. Lanes 2-7 and 8-13 show the ligation products by LIG1 wild-type and E592A mutant obtained at the time points 0.5, 1, 3, 5, 8, and 10 min. *B,* lane 1 is the negative enzyme control of the nicked DNA substrate with 3'-dG opposite template base C. Lanes 2-7 and 8-13 show the ligation products by LIG1 E346A and E346A/E592 mutants obtained at the time points 0.5, 1, 3, 5, 8, and 10 min.

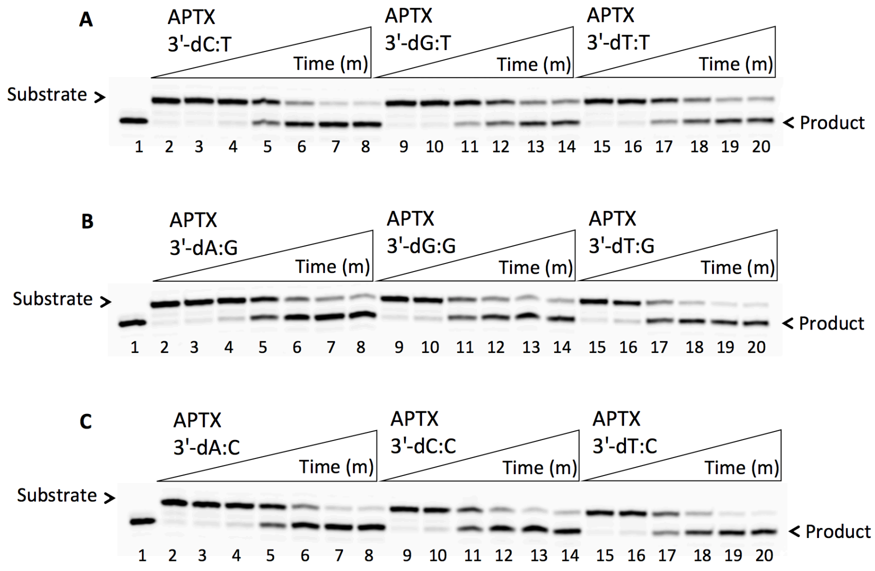

Figure S13. Removal of 5'-AMP by APTX from the ligation failure products with 3'-preinserted mismatches. *A-C,* lane 1 is a size marker that corresponds to the oligonucleotide without an AMP moiety and lane 2 is the negative enzyme control of the nicked DNA substrate with 5'-AMP and 3'-preinserted mismatched base. Lanes 3-8, 9-14, and 15-20 show the products of 5'-AMP removal from 3'-dC:T, 3'-dG:T, and 3'-dT:T (*A*); 3'-dA:G, 3'-dG:G, and 3'-dT:G (*B*); 3'-dA:C, 3'-dC:C, and 3'-dT:C (*C*) mismatch-containing substrates, respectively, obtained at the time points 0.5, 1, 3, 5, 8, and 10 min. The data as the averages from three independent experiments ± SDs are presented in Fig. 15.

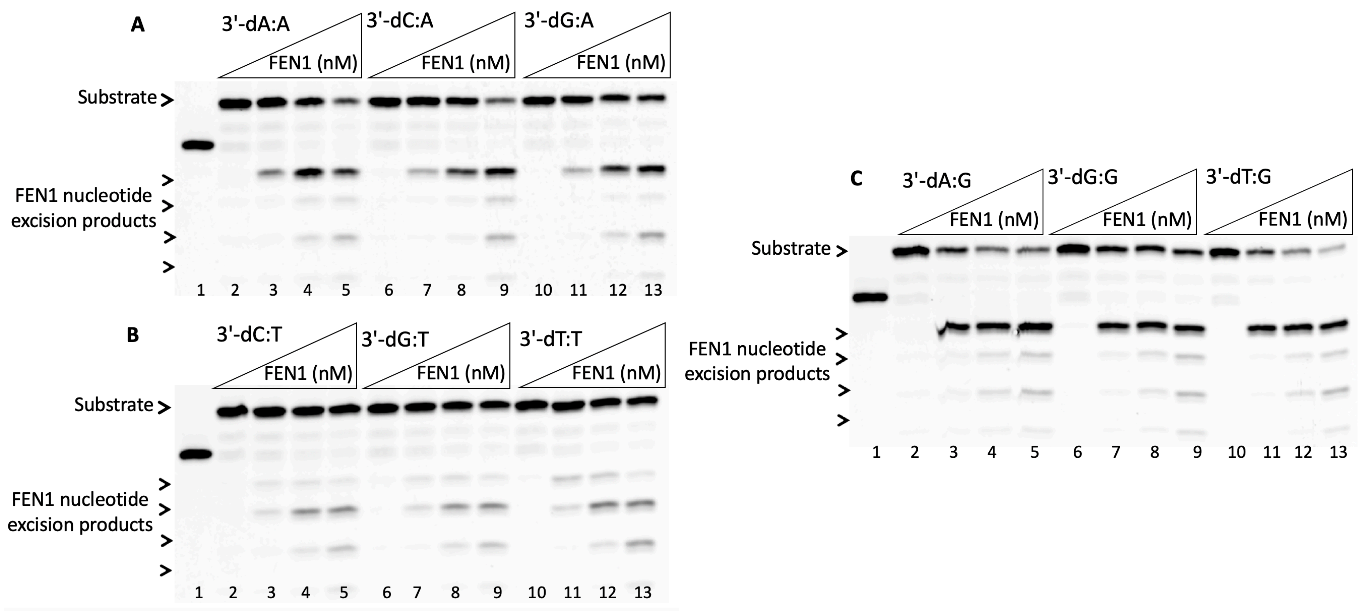

Figure S14. The nucleotide excisions by FEN1 from the ligation failure products with 3'-preinserted mismatches. *A-C,* lane 1 is a size marker that corresponds to the oligonucleotide without an AMP moiety. Lanes 2, 6, and 10 are the negative enzyme controls of the nicked DNA substrates with 5'-AMP and 3'-dA:A, 3'-dC:A, and 3'-dG:A (*A*); 3'-dC:T, 3'-dG:T, and 3'-dT:T (*B*); 3'-dA:G, 3'-dG:G, and 3'-dT:G (*C*) mismatches. Lanes 3-5, 7-9, and 11-13 show the products of nucleotide excisions from the mismatch-containing substrates, respectively, obtained as a function of FEN1 concentration. The data as the averages from three independent experiments ± SDs are presented in Fig. 16.

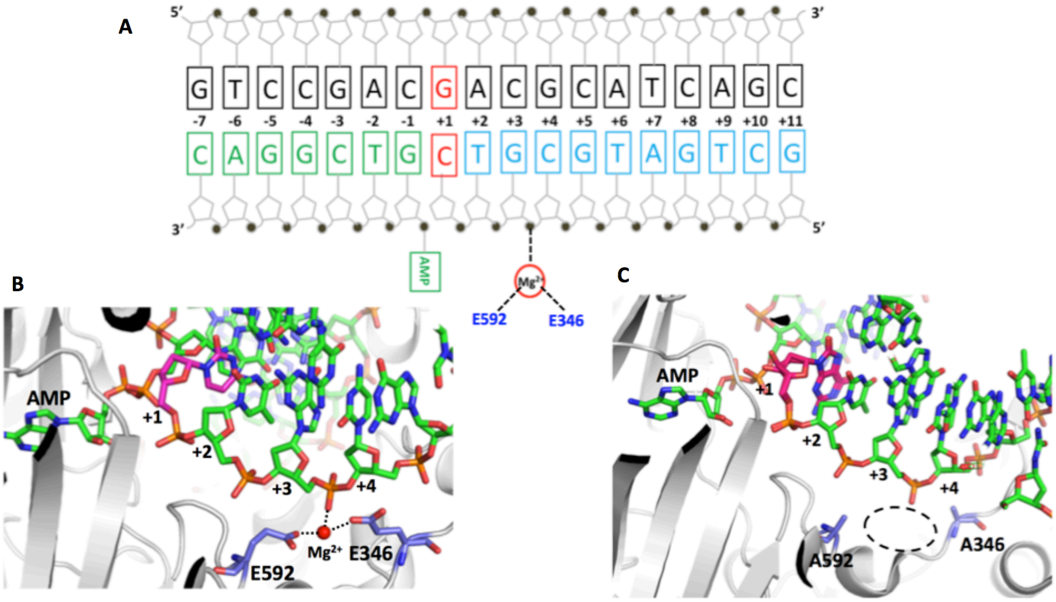

Figure S15. The structure model of LIG1 showing the interactions between high fidelity (HiFi) side chains, Mg^{2+}, and nicked DNA. *A,* nucleotide-residue contact map showing the positions of Mg^{2+} binding site (red), E346 and E592 residues (blue) in complex with the nicked DNA (green) containing G:C base pair. *B,C,* Mg^{HiFi} site interacts with phosphate group between 3rd and 4th base pair of nicked DNA and the sidechains of E592 and E346 residues by salt bond (*B*). The mutations at E346 and E592 to Alanines (E346A/E592A or EE/AA) creates the ligase with lower fidelity due to the Mg^{HiFi} binding site that moves away (dashed circle lines) at the LIG1 active site (*C*). Ribbon diagrams shows LIG1 in contact with a nicked DNA duplex shown as sticks. Structure analysis was performed based on the crystal structure of human LIG1 (PDB:6P0E).

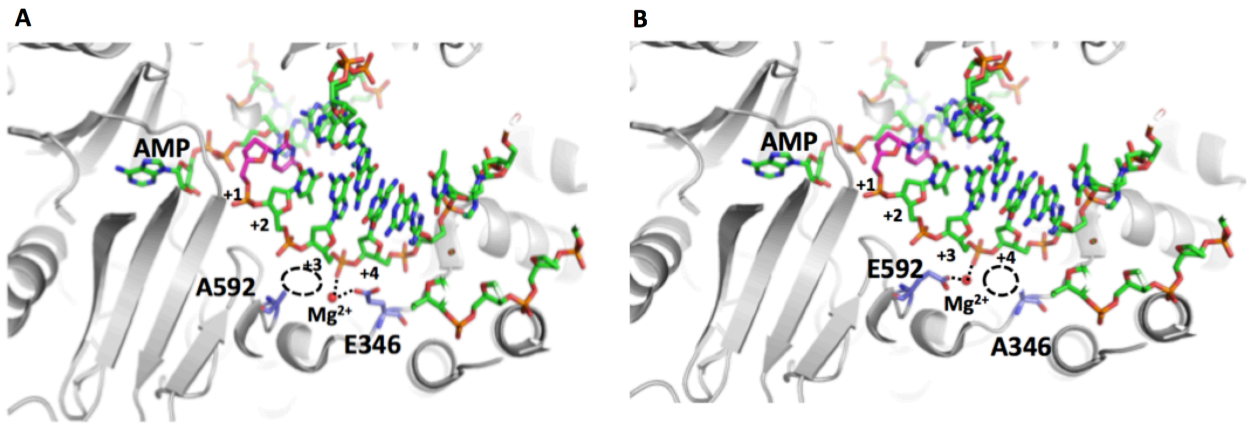

Figure S16. The structure model of LIG1 showing the changes in case of single amino acid substitutions at one of Mg^{HiFi} sites (E346 or E592). When E346 (*A*) or E592 (*B*) are mutated to alanine as shown for E346A (*A*) and E592A (*B*), Mg^{HiFi} would move and forms a little cavity (dashed circle lines) that is smaller than the cavity of the double mutation (E346A/E592A or EE/AA) as shown at the structure model presented in Supplementary Figure 13. Ribbon diagram shows LIG1 (grey) encircling the nicked DNA (sticks) and LIG1 low fidelity site (magenta) are represented by sticks. Structure analysis was performed based on the crystal structure of human LIG1 (PDB:6P0E). Structure model of LIG1 single mutants E592A (*A*) and E346A (*B*).

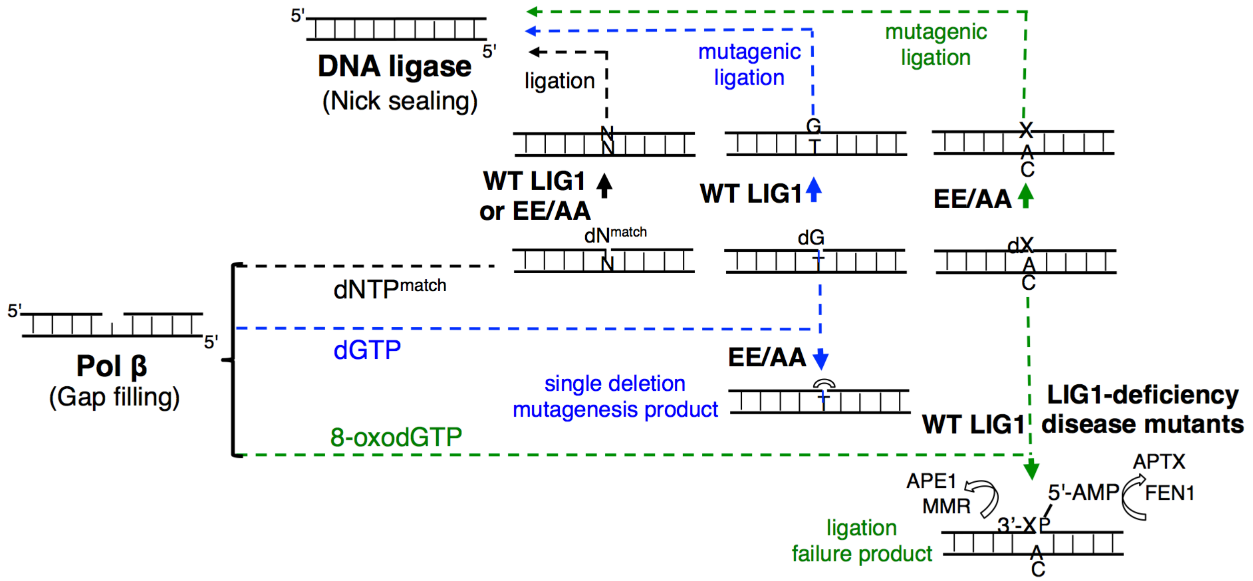

Scheme S1. Illustration of the coordination between pol β and LIG1 during substrate-product channeling at the downstream steps of the BER pathway in the presence of aberrant LIG1 function due to the mutations in the high-fidelity sites (Mg^{HiFi}) or variants associated with LIG1 deficiency disease. The model shows the BER outcomes which could serve as a fidelity checkpoint where the channeling of the repair intermediate could lead to the repair products of a complete ligation ($dNTP^{match}$), single nucleotide deletion mutagenesis (dGTP), mutagenic ligation or ligation failure (8-oxodGTP) from pol β gap filling to the subsequent DNA ligation step by LIG1 wild-type, the low fidelity mutant (E346A/E592A or EE/AA), or LIG1 deficiency disease-associated mutants (P529L, R641L, and R771W).

Substrate	Sequence
Gap Template A	5'-FAM-CATGGGCGGCATGAACC GAGGCCCATCCTCACC-FAM-3'<br>3'-GTACCCGCCGTACTTGGACTCCGGGTAGGAGTGG-5'
Gap Template T	5'-FAM-CATGGGCGGCATGAACC GAGGCCCATCCTCACC-FAM-3'<br>3'-GTACCCGCCGTACTTGGTCTCCGGGTAGGAGTGG-5'
Gap Template C	5'-FAM-CATGGGCGGCATGAACC GAGGCCCATCCTCACC-FAM-3'<br>3'-GTACCCGCCGTACTTGGCCTCCGGGTAGGAGTGG-5'

Table S1. The one nucleotide gapped DNA substrates used in the coupled assays. FAM denotes a flourescence tag and is located at both 5'- and 3'-ends of DNA substrates. The base at template base position is underlined.

Oligonucleotide	Sequence (5'-3')
Template C	GGTGAGGATGGGCCTCCGGTTCATGCCGCCCATG
Template T	GGTGAGGATGGGCCTCTGGTTCATGCCGCCCATG
Template A	GGTGAGGATGGGCCTCAGGTTCATGCCGCCCATG
Template G	GGTGAGGATGGGCCTCGGGTTCATGCCGCCCATG
Downstream primer	(P)GAGGCCCATCCTCACC-FAM
3'-dC	CATGGGCGGCATGAACCC
3'-dG	CATGGGCGGCATGAACCG
3'-dA	CATGGGCGGCATGAACCA
3'-dT	CATGGGCGGCATGAACCT
3'-8-oxodG	CATGGGCGGCATGAACCXdG

Table S2. The sequence of the oligonucleotides used to prepare the nicked DNA substrates for DNA ligation assays. The substrates include 3'-preinserted bases (dA, dT, dG, or dC) or 8-oxodG opposite template base A, T, G, or C. FAM denotes a flourescence tag and P is for a phosphate group. The bases at template base position are underlined.

Oligonucleotide	Sequence (5'-3')
Template C	GGTGAGGATGGGCCTCCGGTTCATGCCGCCCATG
Template T	GGTGAGGATGGGCCTCTGGTTCATGCCGCCCATG
Template A	GGTGAGGATGGGCCTCAGGTTCATGCCGCCCATG
Template G	GGTGAGGATGGGCCTCGGGTTCATGCCGCCCATG
Downstream primer	App-GAGGCCCATCCTCACC-FAM
3'-dC	CATGGGCGGCATGAACCC
3'-dG	CATGGGCGGCATGAACCG
3'-dA	CATGGGCGGCATGAACCA
3'-dT	CATGGGCGGCATGAACCT
3'-8-oxodG	CATGGGCGGCATGAACCXdG

Table S3. The sequence of the oligonucleotides used to prepare the nicked DNA substrates for APTX and FEN1 assays. The substrates include 5'-adenylate or AMP (#) and 3'-preinserted bases (dA, dT, dG, or dC) or 8-oxodG. FAM denotes a flourescence tag and P is for a phosphate group. The bases at template base position are underlined.

Substrate	Sequence
Gap Template T	5'-CATGGGCGGCATGAACC GAGGCCCATCCTCACC-3'#<br>3'-GTACCCGCCGTACTTGGTCTCCGGGTAGGAGTGG-5'

Table S4. The one nucleotide gapped DNA substrate used in the biolayer interferometry assays to analyze DNA binding affinity of LIG1 wild-type and EE/AA mutant. The base at template base position is underlined and # denotes a biotin label at 3'-end.
